# The high-risk phenotype for gastrointestinal vulnerability in sepsis and 28-day mortality: an integrative study based on clinical association and cross-level biological support

**DOI:** 10.3389/fmed.2026.1854067

**Published:** 2026-06-30

**Authors:** Congcong Qin, Weiwei Wang, Qinyuan Du, Li Kong, Batejin, Shuanglin Zhang, Guochen Li

**Affiliations:** 1 Institute of Chinese Medical Literature and Culture, Shandong University of Traditional Chinese Medicine, Jinan, Shandong, China; 2 First Clinical Medical College, Shandong University of Traditional Chinese Medicine, Jinan, Shandong, China; 3 Emergency and Critical Care Medicine Research Center, Affiliated Hospital of Shandong University of Traditional Chinese Medicine, Jinan, Shandong, China; 4 Department of Emergency and Critical Care Medicine, Inner Mongolia International Mongolian Hospital, Hohhot, Inner Mongolia, China

**Keywords:** 28-day mortality, electronic health record phenotype, gastrointestinal vulnerability, GIVP high-risk, MIMIC-IV, reduced external validation, sepsis, single-cell transcriptomics

## Abstract

**Background:**

Gastrointestinal dysfunction is common in sepsis but remains insufficiently represented in organ-specific risk stratification, partly because bedside gastrointestinal findings are often incompletely captured in structured electronic health record data. We defined gastrointestinal vulnerability phenotype (GIVP) high-risk as a prespecified rule-based electronic health record-operationalized phenotype of early gastrointestinal vulnerability and evaluated its prognostic relevance, incremental risk-stratification value, external prognostic directionality, and cross-level biological plausibility.

**Methods:**

This retrospective integrative study used intensive care unit (ICU) admission events with sepsis from the medical information mart for intensive care IV (MIMIC-IV) to construct GIVP high-risk from three prespecified binary domains reflecting early hemodynamic support or hypoperfusion, absence of early enteral nutrition initiation, and early iatrogenic exposure burden. Multivariable logistic regression was used to evaluate the association between GIVP high-risk and fixed-window 28-day mortality. Supportive exposure analyses, sequential organ failure assessment (SOFA)-based stratified analyses, unit-of-analysis sensitivity analyses, Cox sensitivity analysis, incremental prediction analysis, decision curve analysis, and reduced external validation in the eICU collaborative research database (eICU-CRD) were performed. Cross-level biological plausibility was explored using publicly available peripheral blood single-cell transcriptomic data and animal intestinal tissue transcriptomic data.

**Results:**

The primary analysis cohort included 28,224 ICU admission events, of which 6,862 resulted in 28-day mortality. In the core model adjusted for age, sex, Charlson Comorbidity Index, infection source, the continuous SOFA score, and renal replacement therapy, GIVP high-risk retained an adjusted association with fixed-window 28-day mortality (OR = 1.216, 95% CI 1.143–1.295; *P* < 0.001). This association remained directionally consistent after additional adjustment for mechanical ventilation, in unit-of-analysis sensitivity analyses, and in Cox sensitivity analysis, although proportional hazards diagnostics suggested possible non-proportionality. Incremental prediction analysis showed only a very small discrimination improvement in the full cohort, which did not reach DeLong statistical significance, whereas the incremental signal was more concentrated in the high-SOFA subgroup. Decision curve analysis showed small net-benefit gains after adding GIVP high-risk, with maximum ΔNB values of 0.00291266 in the full cohort and 0.00474649 in the high-SOFA subgroup. In eICU-CRD, the reduced GIVP proxy was associated with hospital mortality after adjustment for age, sex, and Acute Physiology and Chronic Health Evaluation (APACHE) score (OR = 1.217, 95% CI 1.183–1.252; *P* < 0.001), supporting external prognostic directionality rather than exact replication of the full MIMIC-IV phenotype. Cross-level analyses showed directional concordance with an interferon (IFN)-high host-response pattern and intestinal tissue transcriptomic patterns characterized by enhanced inflammation, defense imbalance, and abnormal tissue remodeling.

**Conclusion:**

GIVP high-risk is a rule-based electronic health record-operationalized phenotype that retained a prognostic association with 28-day mortality in sepsis after adjustment for clinical context and severity-related variables. Its incremental value was small and context-dependent, with the signal being more evident in higher-severity settings. Reduced external validation supported external prognostic directionality, whereas cross-level transcriptomic analyses supported biological plausibility; neither implied exact external replication nor causal inference.

## 1 Introduction

Sepsis is a life-threatening organ dysfunction caused by an infection-induced dysregulation of the host response and is characterized by marked clinical heterogeneity (1, 2). Previous studies have primarily focused on dimensions such as the source of infection, circulatory failure, immune dysregulation, and organ support pathways (1, 2). However, although the gastrointestinal system is frequently involved during intensive care unit (ICU) care for sepsis, it has long been insufficiently incorporated into organ-specific risk stratification frameworks (3, 4). Compared with organs such as the heart, lungs, and kidneys, gastrointestinal abnormalities in critically ill patients often show more dispersed clinical manifestations and are therefore more easily obscured by overall illness severity (3–5). Existing evidence suggests that acute gastrointestinal injury and gastrointestinal dysfunction are common in critically ill patients and are associated with worse outcomes, including higher mortality risk (3, 4, 6, 7). Current research on gastrointestinal involvement in sepsis has mainly focused on acute gastrointestinal injury grading, feeding intolerance, gastric retention, abnormal intra-abdominal pressure, or single nutritional support strategies (3–8). The ESICM Acute Gastrointestinal Injury grading framework classifies gastrointestinal injury into grades I–IV according to bedside clinical findings, but these findings are often incompletely recorded or inconsistently structured in large electronic health record datasets. Although these indicators are clinically informative, most of these indicators capture only one aspect of gastrointestinal involvement and do not adequately reflect the composite vulnerability state shaped by early hypoperfusion, delayed nutritional implementation, and intensive support-related exposure during the early ICU phase (3–5). In real-world practice, gastrointestinal risk rarely presents as a single isolated abnormality; rather, it more often appears as an aggregated early-risk state reflected by hemodynamic stress, delayed nutritional implementation, and support-related exposure. On this basis, we defined gastrointestinal vulnerability as a rule-based electronic health record-operationalized construct that summarizes early hemodynamic stress or hypoperfusion, delayed or absent early enteral nutrition, and early iatrogenic exposure burden during ICU care in sepsis, with GIVP high-risk serving as the core dichotomous phenotype. This construct is not intended to replace acute gastrointestinal injury grading or direct bedside gastrointestinal assessment; instead, it provides a scalable phenotype for retrospective and multi-database research under structured electronic health record constraints.

For this construct to be clinically and biologically interpretable, several issues must be addressed. First, the source of infection influences organ involvement patterns, host responses, supportive care trajectories, and outcomes in sepsis; therefore, the association between GIVP high-risk and mortality should be evaluated after adjustment for infection background and severity-related factors (2, 8). Second, the additional risk-stratification information provided by GIVP high-risk requires cautious assessment, because this phenotype may partly overlap with global severity and organ-support burden. Third, peripheral host-response patterns do not directly represent local intestinal tissue alterations; therefore, tissue-level transcriptomic evidence may strengthen biological plausibility but cannot establish patient-level causal inference (4, 8, 9). Against this background, the present study used ICU admission events with sepsis in the Medical Information Mart for Intensive Care IV (MIMIC-IV) (10) to construct the high-risk phenotype for gastrointestinal vulnerability and evaluate its association with 28-day mortality. We further examined its robustness, incremental risk-stratification information, and external prognostic directionality through stratified analyses, internal incremental prediction analyses, decision curve analysis, and reduced validation in the eICU Collaborative Research Database (eICU-CRD) (11), while integrating publicly available peripheral blood single-cell data and animal intestinal tissue transcriptomic data to explore cross-level biological plausibility and directional concordance. Overall, this study addressed three related questions: whether this phenotype retains a prognostic association after adjustment for clinical context and severity-related variables, whether its additional information is concentrated in higher-severity settings, and whether it shows directional concordance with host-response and intestinal tissue-level biological patterns.

## 2 Methods

### 2.1 Study design and overall framework

This retrospective integrative analysis evaluated the association between a rule-based electronic health record (EHR)-operationalized phenotype of gastrointestinal vulnerability in sepsis, centered on GIVP high-risk, and 28-day mortality, and examined its robustness, incremental risk-stratification value, reduced external validation, and cross-level interpretability (12–15). The study was organized into four interconnected analytical components: the clinical phenotype construction and association component, the robustness and methodological sensitivity analysis component, the incremental prediction and reduced external validation component, and the cross-level biological interpretability component. The clinical phenotype construction and association component was based on ICU admission events with sepsis from critical care databases and was used to operationally define GIVP high-risk, GIVP score, and supportive component measures and to evaluate their associations with 28-day mortality. The incremental prediction and reduced external validation component was used to examine the incremental risk-stratification value of GIVP high-risk beyond a baseline clinical model, using discrimination, calibration, reclassification, bootstrap validation, and decision curve analysis (DCA), as well as the directionality of a reduced GIVP proxy in eICU-CRD (12, 13, 16). The cross-level biological interpretability component was used to assess whether peripheral host-response and animal tissue transcriptomic findings showed directional concordance with the clinical phenotype, without implying patient-level matched clinical-omics analysis or causal inference (17–23).

### 2.2 Data sources and units of analysis

The data sources comprised three data layers: the MIMIC-IV primary clinical analysis layer, the eICU-CRD reduced external validation layer, and the cross-level biological interpretability layer. The MIMIC-IV primary clinical analysis layer was derived from the curated MIMIC-IV analysis cohort and was used for phenotype construction, main model analysis, supportive exposure analyses, stratified analyses, robustness analyses, and internal incremental prediction and decision curve analyses; the unit of analysis was the ICU admission event. Because some subjects contributed more than one ICU admission event, subject-level duplication was further evaluated in unit-of-analysis sensitivity analyses, including first-ICU restriction, subject-level cluster-robust inference, exchangeable-correlation generalized estimating equations (GEE), and repeated random single-record selection. The eICU-CRD reduced external validation layer used the independent eICU-CRD critical care database to examine the directionality of an electronic health record (EHR)-available reduced GIVP proxy in relation to hospital mortality. Because several full GIVP components, source variables, and original time-window definitions could not be fully reconstructed in eICU-CRD with one-to-one correspondence to the MIMIC-IV definition, reduced external validation, rather than full external replication, was performed using eICU-CRD. A full-to-reduced mapping table and an internal MIMIC-IV reduced-vs-full bridge audit were generated to clarify the relationship between the full GIVP phenotype and the reduced GIVP proxy in eICU-CRD. The domain-level full-to-reduced mapping logic, including partially mappable and unmappable components, is provided in Supplementary Table S1. The cross-level biological interpretability layer included the publicly available peripheral blood single-cell transcriptomic dataset GSE279452 and the external bulk transcriptomic datasets GSE65682 and GSE134347 for host-response analysis, as well as the publicly available animal intestinal tissue transcriptomic datasets GSE202261 and GSE224127 for tissue-module analysis (17–23). Accordingly, MIMIC-IV, eICU-CRD, and public transcriptomic datasets served distinct roles in primary clinical analysis, reduced external validation, and cross-level biological interpretability, respectively.

### 2.3 Definition of study population

An ICU admission-event cohort was first constructed from the MIMIC-IV primary clinical database for sepsis outcome analysis. From the ICU admission events eligible for outcome assessment, gastrointestinal vulnerability-related exposures, sources of infection, overall severity indicators, and other covariates were extracted to form the primary analysis cohort (12–15). The primary analysis included ICU admission events that had complete information for modeling, with the unit of analysis defined as the individual ICU admission episode rather than the unique subject identifier. Because a single subject could contribute more than one ICU admission event, unit-of-analysis sensitivity analyses were conducted to evaluate the impact of the event-level analytical strategy on the primary conclusions, including first-ICU restriction, subject-level cluster-robust inference, exchangeable-correlation generalized estimating equations, and repeated random single-record selection. For the eICU reduced external validation layer, the same event-level principle was applied, with ICU admission events that could serve as complete reduced external validation units included as analysis units for hospital mortality analysis. In the single-cell and animal-level components of the cross-level biological interpretability analysis, the units of analysis were host-response states and tissue transcriptomic modules, respectively, and these data were not incorporated into the clinical main model or used for patient-level causal inference.

### 2.4 Primary outcome, covariates, and adjustment variables

The primary outcome was 28-day all-cause mortality, analyzed as a binary outcome within a fixed time window (12–16). Multivariable logistic regression was used as the primary inferential framework for the fixed-window 28-day mortality analysis. Core covariates included age, sex, Charlson Comorbidity Index, source of infection, the continuous SOFA score, and renal replacement therapy (RRT); among these, source of infection was prespecified as a key clinical confounder, and the continuous SOFA score was derived from the early post-ICU-admission SOFA assessment and used as the primary severity-adjustment variable in the core model (24). Lactate was not included in the core adjustment set because it overlapped with the hypoperfusion component of GIVP and proximal metabolic severity; instead, lactate adjustment was evaluated as an overadjustment sensitivity analysis. Mechanical ventilation was evaluated in an additional sensitivity model to assess whether the association was explained by early ventilatory support. The remaining covariates and auxiliary variables were extracted according to predefined temporal windows and were used for stratified analyses, robustness analyses, overadjustment sensitivity analyses, and evaluation of GIVP-extended models.

### 2.5 Concept of gastrointestinal vulnerability and definition of related exposures

#### 2.5.1 Conceptual definition

Gastrointestinal vulnerability was defined as a rule-based EHR-operationalized clinical construct characterized by gastrointestinal stress, constraints on nutritional implementation, and intensified supportive care pathways during the early phase of ICU admission in patients with sepsis. This construct does not represent a single biological entity and does not replace acute gastrointestinal injury (AGI) grading or single bedside indicators of gastrointestinal function; rather, it operationally summarizes the complex vulnerability profile of the gastrointestinal system in critical care settings (12–15).

#### 2.5.2 Operational definition of GIVP component domains

The GIVP phenotype was constructed from three prespecified binary component domains, with ICU admission used as the temporal anchor. Domain A represented early hemodynamic support or hypoperfusion and was assigned a value of 1 when vasoactive agent exposure was identified within the first 24 h after ICU admission or when the maximum lactate level within the first 24 h was 4 mmol/L or higher; otherwise, it was assigned a value of 0. Domain B represented absence of early enteral nutrition initiation and was assigned a value of 1 when no qualifying enteral nutrition initiation event was identified within the first 72 h after ICU admission; otherwise, it was assigned a value of 0. Domain C represented early iatrogenic exposure burden and was assigned a value of 1 when the total fluid volume within the first 24 h was 2,000 mL or higher, or when invasive mechanical ventilation combined with sedative or opioid exposure was identified within the first 24 h after ICU admission; otherwise, it was assigned a value of 0.

The GIVP score was calculated as the sum of Domain A, Domain B, and Domain C and therefore ranged from 0 to 3. GIVP high-risk was defined as a GIVP score of 2 or higher. AB sum was calculated as the sum of Domain A and Domain B and was used as a supportive two-domain burden measure. The component domains and derived variables were assigned using prespecified rule-based EHR criteria rather than by clustering, LASSO, random forest, or outcome-driven feature selection. The full operational definition of the gastrointestinal vulnerability phenotype, including component domains, time windows, rule-based EHR criteria, coding rules, and analytic roles, is provided in Table 1, with formula-level validation of the GIVP-derived variables provided in Supplementary Table S2. Additional evidence-based operational source definitions for GIVP component domains and derived variables are provided in Supplementary Table S3.

**Table 1:** Operational definition of the gastrointestinal vulnerability phenotype.

Construct	Clinical meaning	Time window	Rule-based EHR criterion	Coding	Role in GIVP
Domain A	Early hemodynamic support or hypoperfusion	0–24 h after ICU admission	Vasoactive-agent exposure within 24 h or maximum lactate within 24 h ≥4 mmol/L	1 if either criterion was met; otherwise 0	Included in GIVP score and AB sum
Domain B	Absence of early enteral nutrition initiation	0–72 h after ICU admission	No qualifying enteral nutrition initiation event identified within 72 h	1 if early enteral nutrition was not identified; otherwise 0	Included in GIVP score and AB sum
Domain C	Early iatrogenic exposure burden	0–24 h after ICU admission	Total fluid volume within 24 h ≥2,000 mL or invasive mechanical ventilation combined with sedative/opioid exposure within 24 h	1 if either exposure pathway was present; otherwise 0	Included in GIVP score
GIVP score	Composite gastrointestinal vulnerability burden	Composite 24 h and 72 h early ICU windows	Domain A + Domain B + Domain C	Integer score from 0 to 3	Ordinal supportive exposure
GIVP high-risk	Core dichotomous gastrointestinal vulnerability phenotype	Composite 24 h and 72 h early ICU windows	GIVP score ≥ 2	1 if GIVP score ≥ 2; otherwise 0	Primary exposure phenotype
AB sum	Two-domain burden based on hemodynamic/hypoperfusion and nutritional implementation domains	Composite 24 h and 72 h early ICU windows	Domain A + Domain B	Integer score from 0 to 2	Supportive exposure

GIVP was defined as a prespecified rule-based EHR-operationalized phenotype rather than a clustering-, machine-learning-, or outcome-driven feature-selection construct. Domain A, Domain B, and Domain C were coded as binary indicators. GIVP score was calculated as Domain A + Domain B + Domain C, and GIVP high-risk was defined as GIVP score ≥2.

EHR, electronic health record; GIVP, gastrointestinal vulnerability phenotype; ICU, intensive care unit.

#### 2.5.3 Definition and primary phenotypic status of GIVP high-risk

GIVP high-risk served as the primary dichotomous exposure in this study and was defined as a GIVP score of 2 or higher. It was used consistently in the main model, sensitivity analyses, stratified analyses, internal incremental prediction analysis, DCA, and reduced external validation (12–16). This phenotype was used to assess whether an EHR-operationalized high-risk state of gastrointestinal vulnerability retained a prognostic association after adjustment for clinical context and severity-related markers.

#### 2.5.4 Supporting roles of GIVP score and AB sum

In addition to GIVP high-risk, GIVP score and AB sum were included as supportive exposures. GIVP score was used to represent ordinal variation in gastrointestinal vulnerability across the three prespecified component domains, whereas AB sum summarized the two-domain burden contributed by Domain A and Domain B. Neither was treated as a parallel primary phenotype; instead, both were used to support the construct validity and interpretability of gastrointestinal vulnerability from complementary dimensions.

### 2.6 Exposure time window and temporal sequence control

The time of ICU admission was used as the primary temporal anchor. Extraction of all primary exposures, covariates, and stratification variables followed three principles: exposure definitions had to precede outcomes, covariates were extracted from the earliest possible window after ICU admission, and stratification and supportive variables were extracted according to predefined temporal windows anchored to ICU admission (12–16). GIVP-related exposures and supportive measures followed the prespecified 24 h and 72 h early ICU windows described above. The source of infection was included as a fixed background variable, and the continuous SOFA score, as an overall indicator of disease severity, was derived from data collected during the early post-admission severity assessment window (24). Overall, primary exposures, supportive exposures, covariates, and stratification variables were extracted using ICU admission as the common temporal anchor to maintain temporal consistency across models.

### 2.7 Definition of infection source and categorization of sparse categories

Infection source was prespecified as a key clinical adjustment variable (12–15). According to the primary analytical framework, infection sources were categorized as pulmonary infection, urinary tract infection, other or unknown infection source, bloodstream infection, abdominal infection, skin and soft tissue infection, and central nervous system infection. Among these, pulmonary infection, urinary tract infection, other or unknown infection source, bloodstream infection, and abdominal infection were treated as the main prespecified infection source categories and were used in the main regression models and prespecified stratified analyses. Skin and soft tissue infection and central nervous system infection were treated as sparse categories because of their small sample sizes. Sparse categories were retained for descriptive presentation and covariate adjustment only, and were not used for standalone category-specific interpretation.

### 2.8 Definition of other covariates

Other covariates included age, sex, the Charlson Comorbidity Index, the continuous SOFA score, and RRT (24, 25). Age and sex were used to characterize basic demographic features, the Charlson Comorbidity Index reflected the burden of pre-existing comorbidities (25), and the continuous SOFA score reflected the overall degree of organ dysfunction early after ICU admission (24). RRT was included in the core adjustment set to account for early renal support and severe organ-support burden. Mechanical ventilation and lactate were handled separately as sensitivity and overadjustment variables, respectively, as described above, rather than as routine core covariates.

### 2.9 Definition of stratification variables

Predefined stratification variables included SOFA-based severity stratification, Domain B assessability stratification, and their combined stratification. SOFA-based severity stratification was constructed from the same early post-ICU-admission SOFA assessment used for severity adjustment in the core model and was used only for prespecified subgroup analyses, not as the continuous severity-adjustment term in the full-cohort model. This stratification was used to assess whether the association pattern of the primary phenotype differed across levels of overall disease severity (24). Domain B assessability stratification was used to examine the stability of the associations involving the primary phenotype and supportive exposures across different levels of information completeness. Combined stratification was constructed on the basis of SOFA-based severity stratification and Domain B assessability stratification and was used to describe stratified association patterns across different combinations of disease severity and Domain B assessability. All stratification variables were prespecified.

### 2.10 Statistical analysis

#### 2.10.1 Descriptive analysis

Continuous variables were summarized as mean and standard deviation or median with interquartile range according to their distribution, whereas categorical variables were summarized as frequencies and percentages (14, 15). Baseline characteristics were compared primarily according to GIVP high-risk status and included age, sex, Charlson Comorbidity Index, SOFA score, source of infection, RRT, Domain B assessability, GIVP component domains, and 28-day mortality.

#### 2.10.2 Main model and sensitivity analyses

The primary outcome was 28-day all-cause mortality, and multivariable logistic regression was used to assess the association between GIVP high-risk and fixed-window 28-day mortality (12–16). The main model included age, sex, Charlson Comorbidity Index, source of infection, the continuous SOFA score, and RRT. An additional mechanical ventilation-adjusted model was fitted to evaluate whether the association was explained by early ventilatory support. A lactate-overadjusted model was fitted as an overadjustment sensitivity analysis because lactate overlapped with the hypoperfusion component of GIVP and proximal metabolic severity. To account for repeated ICU admission events from the same subject, unit-of-analysis sensitivity analyses included first-ICU restriction, subject-level cluster-robust inference, exchangeable-correlation GEE, and repeated random single-record selection.

#### 2.10.3 Supportive exposure analyses

Using the same core adjustment framework as the main model, we separately assessed the associations of the ordinal GIVP score and AB sum with fixed-window 28-day mortality in supportive exposure analyses.

#### 2.10.4 Stratified analyses

Three types of stratified analyses were prespecified: SOFA-based severity stratification, Domain B assessability stratification, and combined SOFA-based severity and Domain B assessability stratification. Within each stratum, the associations of GIVP high-risk, GIVP score, and AB sum with fixed-window 28-day mortality were assessed separately.

#### 2.10.5 Unadjusted event rate analyses

Unadjusted fixed-window 28-day mortality rates were also calculated across different levels of gastrointestinal vulnerability-related exposures to describe the distribution of crude event rates and risk gradients. These analyses were descriptive and were not used as the primary inferential model.

#### 2.10.6 Robustness analyses and methodological boundaries

To assess the impact of using ICU admission as the unit of analysis on the primary results, unit-of-analysis sensitivity analyses were conducted, including first-ICU restriction, subject-level cluster-robust inference, exchangeable-correlation GEE, and repeated random single-record selection (12–15). Within the main model analysis set, the completeness of the primary exposures, outcome, and covariates was verified; because the complete-case indicator was constant, selection inverse probability weighting was not applied. Time-to-event data availability was audited, and Cox proportional hazards regression was fitted as a supportive sensitivity analysis when survival time and 28-day death status were reconstructable. Because the time-to-event candidate dataset was reconstructed according to survival-time availability and was not identical to the complete-case fixed-window logistic cohort, Cox regression was used only to examine directional consistency and was not used to replace or recalibrate the primary fixed-window inference. Proportional hazards diagnostics and time-stratified event tables were used to evaluate possible non-proportionality. Because proportional hazards diagnostics suggested possible non-proportionality for GIVP high-risk, Cox regression was interpreted only as supportive sensitivity evidence, whereas fixed-window multivariable logistic regression remained the primary inferential framework. Fine-Gray competing-risk models were not fitted because competing discharge and censoring structures could not be standardized across the reconstructed analytic datasets.

### 2.11 Incremental prediction analysis and internal validation

#### 2.11.1 Baseline and GIVP-extended model specifications

To evaluate the additional risk-stratification value of GIVP high-risk, the predictive performance of a baseline clinical model and a GIVP-extended model was compared (12, 13, 16). The baseline model included age, sex, the Charlson Comorbidity Index, source of infection, the continuous SOFA score, and RRT, whereas the GIVP-extended model additionally included GIVP high-risk. Both models used fixed-window 28-day all-cause mortality as the outcome and were compared within the same analytical sample.

#### 2.11.2 Discrimination, calibration, reclassification, bootstrap validation, and DCA

Discriminative performance was evaluated using the area under the receiver operating characteristic curve (AUC), and the difference in AUC between the baseline clinical and GIVP-extended models was compared with the DeLong method (16, 26, 27). Overall prediction error was assessed with the Brier score (16, 28). Calibration was evaluated using the calibration intercept, calibration slope, and calibration tables and curves based on decile groups (16, 29, 30). Reclassification and incremental predictive information were assessed using the net reclassification improvement (NRI) and integrated discrimination improvement (IDI). Internal validation was performed using bootstrap resampling to evaluate the stability of model performance estimates. DCA was performed to compare the net benefit of the baseline clinical model and the GIVP-extended model across the prespecified threshold-probability range of 0.05–0.50, using treat-all and treat-none strategies as reference curves. Because no universally accepted single decision threshold exists for using an EHR-derived gastrointestinal vulnerability phenotype to guide management in sepsis, this threshold range was used as an exploratory range for evaluating potential incremental clinical utility. DCA was conducted in the full cohort and in the high-SOFA subgroup and was interpreted as an assessment of supplementary clinical utility rather than as evidence of standalone predictive performance or direct decision-making based on GIVP high-risk alone.

#### 2.11.3 NRI and IDI analysis

Continuous NRI, categorical NRI, and IDI were calculated separately to evaluate changes in risk stratification after adding GIVP high-risk to the baseline clinical model to form the GIVP-extended model (16, 31). The prespecified risk-category thresholds for categorical NRI were 0.1, 0.2, and 0.3.

#### 2.11.4 Bootstrap internal validation

Internal validation was performed using bootstrap resampling with 200 bootstrap resamples. Apparent AUC, AUC optimism, optimism-corrected AUC, apparent Brier score, Brier score optimism, and optimism-corrected Brier score were calculated (13, 16).

#### 2.11.5 Incremental prediction analysis in the high-SOFA subgroup

Within the predefined high-SOFA subgroup, the baseline clinical and GIVP-extended models were further compared with respect to AUC, Brier score, calibration performance, and DCA (16, 26–31).

### 2.12 eICU reduced external validation and mapping logic

#### 2.12.1 General approach to reduced external validation

To examine the prognostic directionality of an EHR-available reduced GIVP proxy in an external database, reduced external validation was conducted using the independent eICU-CRD critical care database. Because the full GIVP high-risk construction process could not be reconstructed with one-to-one correspondence to the MIMIC-IV definition in eICU-CRD, a reduced GIVP proxy was derived from variables available in that database (12, 13, 16). A full-to-reduced mapping table and an internal MIMIC-IV reduced-vs-full bridge audit were generated to clarify the relationship between the full GIVP phenotype and the reduced GIVP proxy in eICU-CRD.

#### 2.12.2 Construction of the reduced GIVP high-risk proxy

The reduced GIVP high-risk proxy in eICU-CRD consisted of four mappable early clinical indicators: absence of enteral nutrition initiation within 24 h, delayed initiation of enteral nutrition beyond 24 h, exposure to mechanical ventilation within 24 h, and exposure to sedation within 24 h. These indicators were coded as binary variables and summed to generate a total indicator score, while the number of evaluable indicators was also recorded. ICU admission events were classified as reduced GIVP high-risk when at least one assessable indicator was present and the total indicator score was 2 or greater. This reduced GIVP proxy was used only for external directional validation and was not interpreted as a one-to-one replacement for the full three-domain GIVP phenotype.

#### 2.12.3 Reduced external validation model setup and evaluation metrics

In the eICU reduced external validation analysis, the representative baseline model included age, sex, and Acute Physiology and Chronic Health Evaluation (APACHE) score, whereas the GIVP-extended model additionally included the reduced GIVP high-risk proxy. Because infection-source categories could not be harmonized between MIMIC-IV and eICU-CRD with sufficient one-to-one comparability, infection source was not included in the representative eICU adjusted model. APACHE score was used as the primary available severity adjustment variable in eICU-CRD. Both models used hospital mortality as the outcome and were fitted in the model-specific complete-case eICU analysis sample. Association estimates for the reduced GIVP high-risk proxy were reported as odds ratios with 95% confidence intervals and *P* values, and model performance was evaluated using AUC, DeLong test results, Brier score, and calibration performance (16, 26–31). All eICU findings were therefore interpreted within this reduced-validation boundary, with the interpretation boundaries for the reduced external validation and cross-level biological analyses summarized in Supplementary Table S4.

### 2.13 Cross-level biological interpretability analysis

#### 2.13.1 General approach

To enhance the biological interpretability of GIVP high-risk, we integrated findings from the peripheral host-response layer and the animal intestinal tissue-transcriptomic layer (17–23). The aim was to examine whether these data layers showed directional concordance with the clinical phenotype, rather than to establish patient-level causal inference.

#### 2.13.2 Analysis framework for the peripheral host-response layer

The peripheral host-response layer was based on publicly available peripheral blood single-cell transcriptomic data. Host-response patterns were evaluated using cluster/state annotations, sample-level scores, and signature-level results (17–23). Publicly available transcriptomic resources were retrieved through the Gene Expression Omnibus (GEO) (17, 18), and the single-cell analytical workflow followed established principles for large-scale single-cell analysis (19). The interferon (IFN)-high host-response axis was used as the principal interpretive anchor.

#### 2.13.3 Analysis framework for the animal intestinal tissue transcriptomic layer

The animal intestinal tissue transcriptomic layer included two publicly available transcriptomic datasets derived from animal intestinal tissue. Tissue transcriptomic modules were used as the analytical unit, and the findings were summarized as three types of tissue-level transcriptomic patterns: enhanced inflammation, defense imbalance, and abnormal tissue remodeling (17–23).

#### 2.13.4 Boundaries for cross-level result interpretation

Cross-level biological interpretability analysis was intended to provide interpretive evidence, with emphasis on directional concordance and recurring biological themes across data layers. It did not involve patient-level matched clinical–omics analysis and was not designed to support causal inference (12–15).

**Figure 1:**
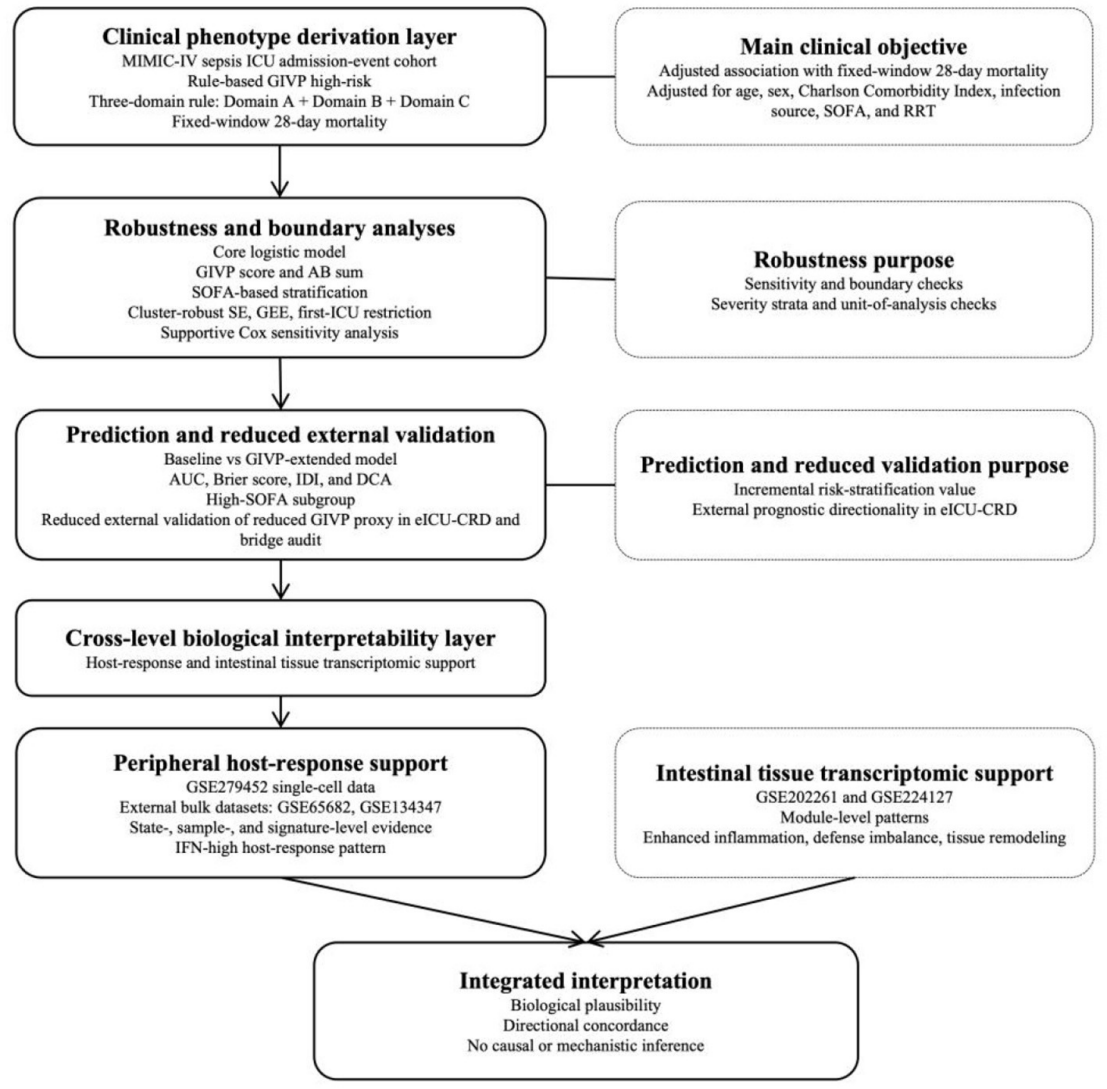
Overall study design and analytical workflow. The study includes four sequential levels: the clinical phenotype derivation layer, robustness and boundary analysis layer, prediction and reduced external validation layer, and cross-level biological interpretability layer. GIVP high-risk was evaluated in relation to fixed-window 28-day mortality, followed by robustness analyses, incremental prediction and decision curve analyses, reduced external validation in eICU-CRD, and cross-level transcriptomic interpretability analyses. AUC, area under the receiver operating characteristic curve; DCA, decision curve analysis; eICU-CRD, eICU Collaborative Research Database; GEE, generalized estimating equation; GIVP, gastrointestinal vulnerability phenotype; IDI, integrated discrimination improvement; IFN, interferon; ICU, intensive care unit; RRT, renal replacement therapy; SOFA, Sequential Organ Failure Assessment.

The overall analytical workflow of this study is shown in Figure 1. The overall framework includes derivation of the primary clinical phenotype, robustness and stratified analyses, internal incremental prediction, reduced external validation in eICU-CRD, and cross-level biological interpretability analyses at the host-response layer and the animal intestinal tissue transcriptomic layer.

## 3 Results

In the primary analysis cohort, the association between GIVP high-risk and fixed-window 28-day mortality was evaluated, followed by prespecified stratified analyses across SOFA-based severity strata. Internal incremental prediction and decision curve analyses, together with reduced external validation of the reduced GIVP proxy in eICU-CRD, were then used to assess the incremental risk-stratification information provided by GIVP high-risk and the external prognostic directionality of the reduced GIVP proxy. Cross-level biological interpretability was further explored by integrating findings from the peripheral host-response layer and the animal intestinal tissue transcriptomic layer.

### 3.1 Cohort composition, baseline characteristics, and GIVP component distribution

The primary analysis cohort included 28,224 ICU admission events, of which 6,862 resulted in fixed-window 28-day mortality, corresponding to a crude mortality rate of 24.31%. When stratified by GIVP high-risk status, the GIVP high-risk negative group comprised 9,273 ICU admission events and the GIVP high-risk positive group comprised 18,951 ICU admission events. Baseline characteristics differed between the two groups. Compared with the negative group, the positive group had a higher proportion of males, higher SOFA scores, and a higher 28-day mortality rate; conversely, the positive group had a lower mean Charlson Comorbidity Index and a lower proportion of Domain B-assessable events. The positive group also showed higher peak lactate levels, more frequent mechanical ventilation, and greater vasoactive agent use. Age and the distribution of RRT did not differ significantly between the two groups. Because infection source was considered a clinically relevant background factor and its overall distribution differed between groups, it was retained as a prespecified covariate in subsequent adjusted models. The baseline characteristics of the primary analysis cohort stratified by GIVP high-risk status are summarized in Table 2.

**Table 2:** Baseline characteristics of the primary analysis cohort stratified by GIVP high-risk status.

Variables	Overall (*n* = 28,224)	GIVP high-risk = 0 (*n* = 9,273)	GIVP high-risk = 1 (*n* = 18,951)	*P* value
Sample size, *n*	28,224	9,273	18,951	–
Age, years	64.16 ± 15.69	63.98 ± 16.04	64.25 ± 15.52	0.259
Charlson Comorbidity Index	5.51 ± 2.99	5.80 ± 3.01	5.36 ± 2.97	< 0.001
SOFA score	3.96 ± 2.18	3.63 ± 2.09	4.12 ± 2.20	< 0.001
Male, *n* (%)	16,603 (58.8%)	5,292 (57.1%)	11,311 (59.7%)	< 0.001
RRT, *n* (%)	3,995 (14.2%)	1,362 (14.7%)	2,633 (13.9%)	0.075
Domain B assessable, *n* (%)	6,348 (22.5%)	3,317 (35.8%)	3,031 (16.0%)	< 0.001
28-day mortality, *n* (%)	6,862 (24.3%)	2,172 (23.4%)	4,690 (24.7%)	0.015
Peak lactate within 24 h, mmol/L	2.98 ± 2.84	1.86 ± 1.04	3.53 ± 3.25	< 0.001
Mechanical ventilation, *n* (%)	16,468 (58.3%)	3,917 (42.2%)	12,551 (66.2%)	< 0.001
Vasoactive agents, *n* (%)	14,785 (52.4%)	1,065 (11.5%)	13,720 (72.4%)	< 0.001
Overall distribution of infection sources	–	–	–	< 0.001
Pulmonary infection, *n* (%)	8,045 (28.5%)	3,315 (35.7%)	4,730 (25.0%)	–
Urinary tract infection, *n* (%)	6,635 (23.5%)	1,944 (21.0%)	4,691 (24.8%)	–
Other or unknown source of infection, *n* (%)	6,159 (21.8%)	1,451 (15.6%)	4,708 (24.8%)	–
Bloodstream infection, *n* (%)	3,795 (13.4%)	1,363 (14.7%)	2,432 (12.8%)	–
Abdominal infection, *n* (%)	3,280 (11.6%)	1,126 (12.1%)	2,154 (11.4%)	–
Skin and soft tissue infection, *n* (%)	271 (1.0%)	56 (0.6%)	215 (1.1%)	–
Central nervous system infection, *n* (%)	39 (0.1%)	18 (0.2%)	21 (0.1%)	–

Continuous variables are expressed as mean ± standard deviation, and categorical variables are expressed as *n* (%). *P* values compare GIVP high-risk positive and negative groups. Domain B source information assessable indicates that the information required to evaluate the enteral-nutrition-related domain could be extracted within the predefined time window and does not indicate Domain B positivity. The *P* value for infection source represents the overall between-group difference across infection source categories.

GIVP, gastrointestinal vulnerability phenotype; SOFA, sequential organ failure assessment.

At the component level, the GIVP high-risk group showed a multi-domain accumulation pattern. In the GIVP low-risk group, Domain A, Domain B, and Domain C were positive in 12.12%, 65.11%, and 12.24% of events, respectively, with a mean GIVP score of 0.8947. In contrast, in the GIVP high-risk group, Domain A, Domain B, and Domain C were positive in 80.74%, 86.17%, and 81.89% of events, respectively, with a mean GIVP score of 2.4880 (Supplementary Table S5).

### 3.2 Primary outcome: association between GIVP high-risk and fixed-window 28-day mortality

In the multivariable logistic regression model with fixed-window 28-day all-cause mortality as the outcome, the core model was adjusted for age, sex, the Charlson Comorbidity Index, source of infection, the continuous SOFA score, and RRT. GIVP high-risk retained an adjusted association with fixed-window 28-day mortality, with an adjusted OR of 1.216 (95% CI 1.143–1.295; *P* < 0.001).

After further adjustment for mechanical ventilation, the association between GIVP high-risk and fixed-window 28-day mortality persisted, with an adjusted OR of 1.169 (95% CI 1.096–1.246; *P* < 0.001). In the lactate-overadjusted model, the estimate reversed direction (OR 0.782, 95% CI 0.731–0.836; *P* < 0.001), which was interpreted as overadjustment because lactate overlapped with the hypoperfusion component of GIVP and proximal metabolic severity. Based on these findings, GIVP high-risk was carried forward to supportive exposure analyses, SOFA-based severity stratification, incremental prediction and decision curve analyses, and reduced external validation. The primary association estimates and representative sensitivity models for GIVP high-risk and fixed-window 28-day mortality are summarized in Table 3.

**Table 3:** Primary association and sensitivity models for GIVP high-risk and fixed-window 28-day mortality.

Analysis	Adjustment	Effect measure	Estimate	95% CI	*P* value	AUC	Interpretation
Crude model	None	OR	1.075	1.014–1.140	0.015	0.508	Unadjusted association
Model 1	Age, sex, Charlson Comorbidity Index, infection source	OR	1.276	1.200–1.356	< 0.001	0.684	Adjusted for demographic, comorbidity, and infection background
Model 2	Model 1 + continuous SOFA score	OR	1.210	1.137–1.287	< 0.001	0.690	Adjusted for broad severity marker
Model 3/core model	Model 2 + RRT	OR	1.216	1.143–1.295	< 0.001	0.706	Final primary association model
Mechanical ventilation sensitivity model	Core model + mechanical ventilation	OR	1.169	1.096–1.246	< 0.001	0.706	Directionally consistent after ventilatory-support adjustment
Lactate overadjustment sensitivity model	Core model + lactate	OR	0.782	0.731–0.836	< 0.001	0.756	Direction reversal suggesting overadjustment

The core model adjusted for age, sex, Charlson Comorbidity Index, infection source, the continuous SOFA score, and RRT. The mechanical ventilation model was used as an additional sensitivity analysis. The lactate-overadjusted model was interpreted as an overadjustment sensitivity analysis because lactate overlapped with the hypoperfusion component of GIVP and proximal metabolic severity.

AUC, area under the receiver operating characteristic curve; CI, confidence interval; GIVP, gastrointestinal vulnerability phenotype; OR, odds ratio; RRT, renal replacement therapy; SOFA, sequential organ failure assessment.

### 3.3 Results on supportive exposures and SOFA-based severity stratification

#### 3.3.1 Supportive results for GIVP score and AB sum

In the primary analysis cohort, after adjustment for age, sex, the Charlson Comorbidity Index, infection source, the continuous SOFA score, and RRT, GIVP score remained associated with fixed-window 28-day mortality, with an OR of 1.085 (95% CI 1.048–1.122; *P* < 0.001). AB sum also remained associated with fixed-window 28-day mortality, with an OR of 1.262 (95% CI 1.202–1.325; *P* < 0.001).

#### 3.3.2 Results by SOFA-based severity stratification

After stratification according to SOFA-based severity, the association between GIVP high-risk and fixed-window 28-day mortality was more evident in the high-SOFA subgroup. In the high-SOFA subgroup, GIVP high-risk remained associated with fixed-window 28-day mortality (OR 1.285, 95% CI 1.193–1.384; P < 0.001). In contrast, this association did not reach statistical significance in the low-SOFA subgroup (OR 1.066, 95% CI 0.948–1.197; *P* = 0.2862).

A similar pattern was observed for the supportive exposures. In the high-SOFA subgroup, GIVP score was associated with fixed-window 28-day mortality (OR 1.115, 95% CI 1.072–1.160; *P* < 0.001), and AB sum also remained associated with fixed-window 28-day mortality (OR 1.317, 95% CI 1.244–1.394; *P* < 0.001). In the low-SOFA subgroup, GIVP score was not associated with fixed-window 28-day mortality (OR 0.998, 95% CI 0.932–1.069; *P* = 0.9515), whereas AB sum retained a modest association (OR 1.110, 95% CI 1.007–1.223; *P* = 0.0358). The results of the supportive exposure analyses and SOFA-based severity stratification are summarized in Table 4.

**Table 4:** Supportive exposure analyses and SOFA-based severity stratification for fixed-window 28-day mortality.

Analysis stratum	Exposure	Effect measure	Estimate	95% CI	*P* value	Adjustment
Full cohort	GIVP high-risk	OR	1.216	1.143–1.295	< 0.001	Age, sex, Charlson Comorbidity Index, infection source, continuous SOFA score, RRT
Full cohort	GIVP score	OR	1.085	1.048–1.122	< 0.001	Age, sex, Charlson Comorbidity Index, infection source, continuous SOFA score, RRT
Full cohort	AB sum	OR	1.262	1.202–1.325	< 0.001	Age, sex, Charlson Comorbidity Index, infection source, continuous SOFA score, RRT
Low-SOFA subgroup	GIVP high-risk	OR	1.066	0.948–1.197	0.2862	Age, sex, Charlson Comorbidity Index, infection source, RRT
Low-SOFA subgroup	GIVP score	OR	0.998	0.932–1.069	0.9515	Age, sex, Charlson Comorbidity Index, infection source, RRT
Low-SOFA subgroup	AB sum	OR	1.110	1.007–1.223	0.0358	Age, sex, Charlson Comorbidity Index, infection source, RRT
High-SOFA subgroup	GIVP high-risk	OR	1.285	1.193–1.384	< 0.001	Age, sex, Charlson Comorbidity Index, infection source, RRT
High-SOFA subgroup	GIVP score	OR	1.115	1.072–1.160	< 0.001	Age, sex, Charlson Comorbidity Index, infection source, RRT
High-SOFA subgroup	AB sum	OR	1.317	1.244–1.394	< 0.001	Age, sex, Charlson Comorbidity Index, infection source, RRT

Full-cohort models were adjusted for age, sex, Charlson Comorbidity Index, infection source, the continuous SOFA score, and RRT. In SOFA-based severity strata, the continuous SOFA score was omitted from the stratified models because the subgroup strata were defined using the SOFA-based severity stratification variable; stratified models were adjusted for age, sex, Charlson Comorbidity Index, infection source, and RRT.

AB sum, sum of Domain A and Domain B; CI, confidence interval; GIVP, gastrointestinal vulnerability phenotype; OR, odds ratio; RRT, renal replacement therapy; SOFA, sequential organ failure assessment.

**Figure 2:**
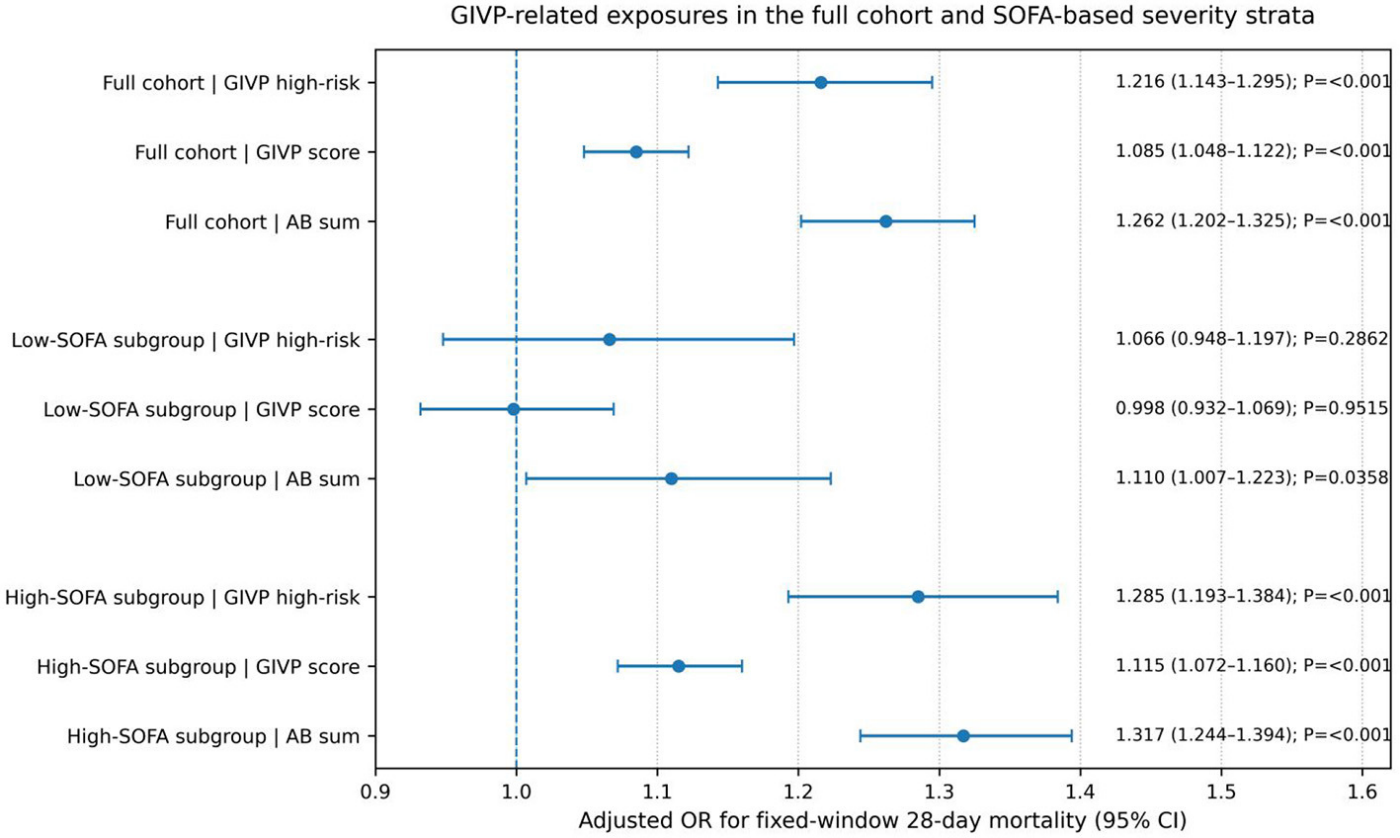
GIVP-related exposures across SOFA-based severity strata. The forest plot presents adjusted ORs and 95% CIs for GIVP high-risk, GIVP score, and AB sum in the full cohort, low-SOFA subgroup, and high-SOFA subgroup. Full-cohort models were adjusted for age, sex, Charlson Comorbidity Index, infection source, the continuous SOFA score, and RRT; stratified models were adjusted for age, sex, Charlson Comorbidity Index, infection source, and RRT. AB sum represents the sum of Domain A and Domain B. The dashed line indicates OR = 1. CI, confidence interval; GIVP, gastrointestinal vulnerability phenotype; OR, odds ratio; RRT, renal replacement therapy; SOFA, sequential organ failure assessment.

The SOFA-stratified exposure estimates are visualized in Figure 2.

### 3.4 Robustness and methodological boundary analyses

#### 3.4.1 Unit-of-analysis and within-patient correlation sensitivity analyses

In the primary analysis dataset, 28,224 ICU admission events corresponded to 23,273 unique subjects, indicating that some patients contributed repeated ICU records. The association between GIVP high-risk and fixed-window 28-day mortality remained directionally consistent across unit-of-analysis sensitivity analyses. In the full dataset using subject-level cluster-robust standard errors, GIVP high-risk retained an adjusted association with fixed-window 28-day mortality (OR 1.216, 95% CI 1.141–1.297; *P* < 0.001). In the GEE logistic model with exchangeable working correlation clustered by subject_id, GIVP high-risk also remained associated with fixed-window 28-day mortality (OR 1.193, 95% CI 1.122–1.269; *P* < 0.001), with an estimated working correlation of 0.2386. In the first-ICU-stay restricted analysis, the association remained directionally consistent (OR 1.195, 95% CI 1.114–1.283; *P* < 0.001). Across 200 repeated random single-record selections, the median OR was 1.230, with an empirical 2.5th−97.5th percentile interval of 1.200–1.254; all valid iterations yielded an OR >1 with *P* < 0.05. These findings indicate that the primary association was not explained by repeated ICU admission events from the same subject.

#### 3.4.2 Completeness of data assessment and selection inverse probability weighting boundaries

There were no missing values for the primary exposure, outcome, or covariates included in the core model. The complete-case sample size was therefore identical to that of the primary analysis cohort, both at 28,224 ICU admission events. Because the complete-case indicator was constant in the analytic dataset, selection inverse probability weighting was not implemented.

#### 3.4.3 Time-to-event sensitivity analysis and proportional hazards boundary

A time-to-event availability audit identified a candidate Cox dataset containing complete survival time, 28-day death event status, and GIVP high-risk data. The usable candidate dataset included 41,295 candidate records with reconstructable survival time, 28-day death status, and GIVP high-risk status, including 8,888 deaths and 32,407 censored observations. There were no missing values in survival time, event status, or GIVP high-risk, and no non-positive survival times or event times beyond the 28-day window were detected. Because this candidate time-to-event dataset was reconstructed according to survival-time availability and was not identical to the complete-case fixed-window logistic cohort, the Cox analysis was used only to examine directional consistency and was not used to replace or recalibrate the primary fixed-window inference.

In Cox sensitivity analyses, GIVP high-risk showed a directionally consistent association with 28-day mortality. In the core adjusted Cox model, GIVP high-risk was associated with higher 28-day mortality risk (HR 1.054, 95% CI 1.010–1.100; *P* = 0.015). However, proportional hazards diagnostics suggested possible non-proportionality for GIVP high-risk. Time-stratified event analysis showed that the excess event rate associated with GIVP high-risk was concentrated in the earliest follow-up interval. During 0–3 days, the event rate was 2.8993 per 100 person-days in the GIVP high-risk group and 0.8339 per 100 person-days in the GIVP low-risk group, corresponding to an event-rate ratio of 3.4768. In later intervals, the event-rate ratio attenuated. Therefore, Cox regression was interpreted as supportive time-to-event sensitivity evidence for directional consistency, rather than as a primary inferential model or a recalibration of the fixed-window logistic analysis. Fine-Gray competing-risk modeling was not performed because competing discharge and censoring structures could not be standardized across the reconstructed analytic datasets; therefore, Fine-Gray results were not reported and fixed-window logistic regression remained the primary analysis. The results of the robustness analyses and methodological boundary assessments are summarized in Table 5.

**Table 5:** Robustness and methodological boundary analyses.

Analysis	Dataset	Effect measure	Estimate	95% CI/interval	*P* value	*n*	Events	Additional information
Subject-level cluster-robust logistic regression	Full dataset	OR	1.216	1.141–1.297	< 0.001	28,224	6,862	Clusters/subjects = 23,273
GEE logistic model with exchangeable working correlation	Full dataset	OR	1.193	1.122–1.269	< 0.001	28,224	6,862	Clusters = 23,273; working correlation alpha = 0.2386
First-ICU-stay restricted logistic regression	First ICU stay per subject	OR	1.195	1.114–1.283	< 0.001	23,273	5,488	One ICU stay retained per subject
Repeated random single-record selection	One randomly selected ICU record per subject across 200 iterations	OR	1.230	1.200–1.254	–	200 iterations	–	Median OR; empirical 2.5th−97.5th percentile interval; 100% iterations OR > 1 and *P* < 0.05
Cox sensitivity analysis	Time-to-event candidate dataset	HR	1.054	1.010–1.100	0.015	41,295	8,888	Interpreted as supportive sensitivity analysis
Proportional hazards diagnostic	Cox sensitivity dataset	Residual-log(time) correlation	−0.278	–	< 0.001	41,295	8,888	Possible non-proportionality for GIVP high-risk
Time-stratified event pattern, 0–3 d	Cox sensitivity dataset	Event-rate ratio	3.477	–	–	41,295	447 vs 1,928	Risk sets: 18,053 in GIVP low-risk and 23,242 in GIVP high-risk; event rates per 100 person-days: 0.8339 vs. 2.8993

Unit-of-analysis sensitivity analyses were used to assess whether repeated ICU records from the same subject influenced the primary association. The Cox sensitivity dataset was reconstructed separately according to survival-time availability and was not identical to the fixed-window logistic primary analysis cohort. Cox regression was interpreted as supportive because proportional hazards diagnostics suggested possible non-proportionality for GIVP high-risk, and fixed-window logistic regression remained the primary inferential model. Fine-Gray competing-risk models were not fitted because competing discharge and censoring structures could not be standardized.

CI, confidence interval; GEE, generalized estimating equation; GIVP, gastrointestinal vulnerability phenotype; HR, hazard ratio; ICU, intensive care unit; OR, odds ratio.

### 3.5 Incremental prediction and decision curve analyses

#### 3.5.1 Incremental prediction performance in the full cohort

In the primary analysis cohort, a baseline prediction model was constructed using age, sex, the Charlson Comorbidity Index, infection source, the continuous SOFA score, and RRT. The GIVP-extended model additionally included GIVP high-risk. The AUC of the baseline model was 0.705088, and that of the GIVP-extended model was 0.705983, corresponding to an AUC difference of 0.000895 (DeLong 95% CI −0.000033 to 0.001824; *P* = 0.0587). This full-cohort AUC increase did not reach conventional statistical significance by DeLong testing. The Brier score decreased from 0.167215 to 0.166851, with a difference of −0.000364. The continuous NRI was 0.031774, the IDI was 0.001744, and the categorical NRI was 0.003634. These results indicated that adding GIVP high-risk produced only a very small improvement in discrimination and prediction error in the full cohort; therefore, the full-cohort incremental discrimination gain should be interpreted as statistically non-significant and clinically modest.

#### 3.5.2 Incremental prediction performance in the High-SOFA subgroup

In the predefined high-SOFA subgroup analysis, a total of 19,547 ICU admission events were included, of which 5,270 resulted in fixed-window 28-day mortality. In this subgroup, the AUC of the baseline model was 0.689033, and that of the GIVP-extended model was 0.690987, with an AUC difference of 0.001954 (DeLong 95% CI 0.000483 to 0.003424; *P* = 0.0092). The Brier score decreased from 0.180831 to 0.180274, with a difference of −0.000557. The continuous NRI was 0.030864, the IDI was 0.002611, and the categorical NRI was 0.007859. Relative to the full cohort, the incremental information provided by GIVP high-risk was more concentrated in the high-SOFA subgroup, although the absolute improvement remained modest.

#### 3.5.3 Decision curve analysis

Decision curve analysis was performed to compare the baseline prediction model with the GIVP-extended model in the full cohort and in the high-SOFA subgroup. In the full cohort, adding GIVP high-risk yielded a small net-benefit gain, with a maximum ΔNB of 0.00291266 and a mean ΔNB of 0.00059828 across the evaluated threshold range. In the high-SOFA subgroup, the corresponding maximum ΔNB was 0.00474649 and the mean ΔNB was 0.00086081. DCA was interpreted over the prespecified exploratory threshold-probability range of 0.05–0.50, because no universally accepted single decision threshold exists for using an EHR-derived gastrointestinal vulnerability phenotype to guide sepsis management. Although adding GIVP high-risk yielded positive net-benefit differences across part of the evaluated threshold range, the absolute magnitude of improvement was small. Therefore, these findings support limited supplementary clinical utility and complementary risk stratification, rather than direct clinical decision-making based on GIVP high-risk alone or use of GIVP high-risk as a clinically decisive prediction model. Table 6 summarizes the incremental prediction and decision-curve results after adding GIVP high-risk to the baseline model in the full cohort and high-SOFA subgroup.

**Table 6:** Incremental prediction and decision-curve analysis after adding GIVP high-risk.

Cohort	Baseline AUC	GIVP-extended AUC	ΔAUC	DeLong *P* value for ΔAUC	ΔBrier	IDI	Maximum ΔNB	Mean ΔNB
Full cohort	0.705088	0.705983	0.000895	0.0587	−0.000364	0.001744	0.00291266	0.00059828
High-SOFA subgroup	0.689033	0.690987	0.001954	0.0092	−0.000557	0.002611	0.00474649	0.00086081

The baseline model included age, sex, Charlson Comorbidity Index, infection source, the continuous SOFA score, and RRT. The GIVP-extended model additionally included GIVP high-risk. ΔAUC and ΔBrier were calculated as the GIVP-extended model minus the baseline model. Decision-curve results were interpreted across the prespecified exploratory threshold-probability range of 0.05–0.50 because no universally accepted single decision threshold exists for using an EHR-derived gastrointestinal vulnerability phenotype to guide sepsis management. The observed net-benefit gains were small in absolute magnitude and therefore supported limited supplementary clinical utility and complementary risk stratification rather than standalone clinical decision-making.

AUC, area under the receiver operating characteristic curve; ΔNB, delta net benefit; GIVP, gastrointestinal vulnerability phenotype; IDI, integrated discrimination improvement; RRT, renal replacement therapy; SOFA, sequential organ failure assessment.

**Figure 3:**
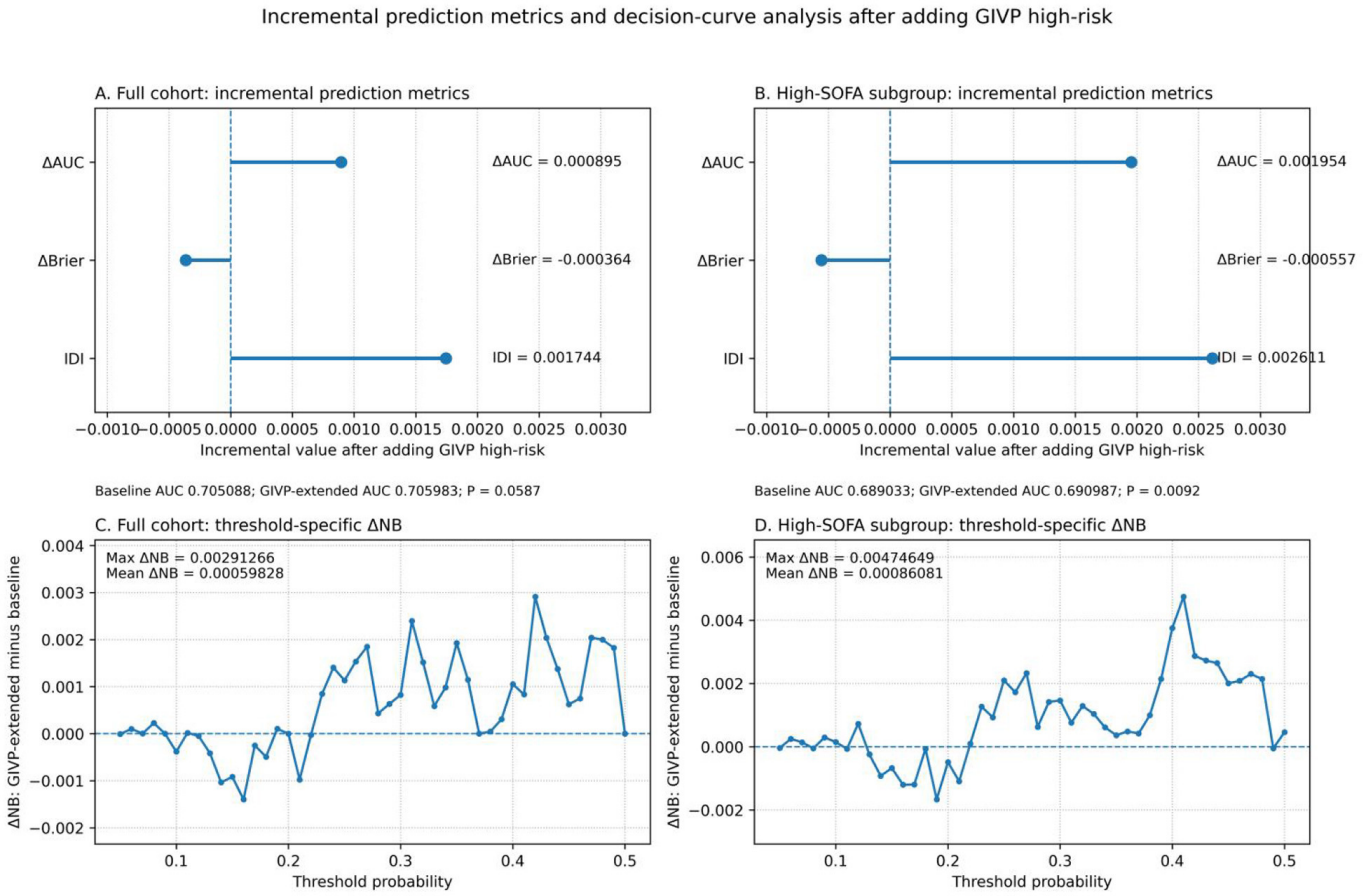
Incremental prediction metrics and decision-curve analysis after adding GIVP high-risk. **(A, B)** Present the incremental changes in model performance after adding GIVP high-risk to the baseline model in the full cohort and high-SOFA subgroup, respectively, including ΔAUC, ΔBrier, and IDI. **(C, D)** Show threshold-specific delta net benefit (ΔNB) across the evaluated threshold-probability range of 0.05–0.50, calculated as the net benefit of the GIVP-extended model minus that of the baseline model. Positive ΔNB values indicate a net-benefit gain after adding GIVP high-risk. AUC, area under the receiver operating characteristic curve; IDI, integrated discrimination improvement; ΔNB, delta net benefit; GIVP, gastrointestinal vulnerability phenotype; SOFA, sequential organ failure assessment.

The incremental prediction metrics and decision-curve results are visualized in Figure 3.

### 3.6 Reduced external validation of the reduced GIVP proxy in eICU-CRD

#### 3.6.1 Composition of the eICU external validation cohort

In the external eICU-CRD database, a reduced external validation cohort was constructed according to cross-database variable availability. A total of 319,503 ICU admission events were included, comprising 29,798 in-hospital deaths and 289,705 survivors.

#### 3.6.2 Distribution of reduced GIVP high-risk

In the eICU reduced validation cohort, component-level inspection showed substantial imbalance in the nutrition-related reduced indicators. Absence of enteral nutrition initiation within 24 h was present in 309,950 of 319,503 ICU admission events (97.0%), whereas delayed enteral nutrition initiation beyond 24 h was not observed as a positive component among evaluable records (0.0%). Mechanical ventilation within 24 h and sedation within 24 h were present in 16,083 (5.0%) and 164,036 (51.3%) ICU admission events, respectively. Therefore, the eICU reduced score mainly reflected variation in mechanical ventilation and sedation on the background of near-universal absence of early enteral nutrition, rather than a balanced reconstruction of the full MIMIC-IV three-domain GIVP phenotype. Overall, 155,667 ICU admission events were classified as having reduced GIVP high-risk = 0, and 163,836 were classified as having reduced GIVP high-risk = 1. Detailed component and score distributions are summarized in Supplementary Table S6.

#### 3.6.3 Full-to-reduced GIVP bridge analysis

Because the full three-domain GIVP definition could not be reconstructed one-to-one in eICU-CRD, the eICU analysis was interpreted as reduced external validation rather than full external replication. A full-to-reduced mapping table was constructed to clarify the relationship between the MIMIC-IV full GIVP construct and the eICU reduced GIVP proxy, with the domain-level mapping logic provided in Supplementary Table S1. In the MIMIC-IV internal bridge audit, the available reduced score was reconstructed using the single eICU-mappable indicator identified in MIMIC-IV (mechanical ventilation), constraining the maximum simulated reduced score to 1; therefore, available reduced score ≥1 was used as the bridge audit threshold. In the actual eICU-CRD analysis, multiple EHR-available reduced indicators were available in eICU-CRD, and a component sum ≥2 threshold was applied. The bridge audit showed partial rather than exact agreement between the reduced proxy and the full GIVP phenotype, with an accuracy of 0.634 and a Cohen kappa of 0.225. Therefore, reduced GIVP high-risk was not interpreted as a direct substitute for the full three-domain GIVP phenotype.

#### 3.6.4 Association and prediction results in eICU-CRD

In eICU-CRD, reduced GIVP high-risk was associated with hospital mortality. In the crude model, this proxy was associated with higher odds of hospital mortality (OR 1.460, 95% CI 1.425–1.496; *P* < 0.001; AUC 0.547; *n* = 319,503). In the representative adjusted model including age, sex, and Acute Physiology and Chronic Health Evaluation (APACHE) score, reduced GIVP high-risk remained associated with hospital mortality (OR 1.217, 95% CI 1.183–1.252; *P* < 0.001; *n* = 296,950; AUC 0.822). The representative adjusted model included 296,950 ICU admission events because APACHE score was required for model fitting; events without available APACHE score were excluded from this model-specific complete-case analysis. Sensitivity models using acute physiology score (APS), with or without APACHE score, showed directionally consistent results. These findings support the external prognostic directionality of the EHR-available reduced GIVP proxy.

#### 3.6.5 Decision curve results in eICU-CRD

Decision curve analysis in eICU-CRD showed a detectable but very small incremental net-benefit gain after adding reduced GIVP high-risk. Across the evaluated threshold-probability range of 0.05–0.50, the maximum ΔNB was 0.00048288 and the mean ΔNB was 0.00009446. Therefore, the eICU decision-curve results were interpreted cautiously across the evaluated threshold-probability range and supported directional transportability rather than strong clinical utility. The reduced external validation models, eICU decision-curve summary, and MIMIC-IV reduced-vs.-full bridge audit are summarized in Table 7.

**Table 7:** Reduced external validation of the reduced GIVP proxy in eICU-CRD and MIMIC-IV bridge audit.

Analysis	Adjustment	Effect measure	Estimate	95% CI	*P* value	*n*	AUC
Crude model	None	OR	1.460	1.425–1.496	< 0.001	319,503	0.547
Representative adjusted model	Age, sex, APACHE score	OR	1.217	1.183–1.252	< 0.001	296,950	0.822
APS sensitivity model	Age, sex, acute physiology score	OR	1.206	1.172–1.241	< 0.001	296,950	0.820
DCA summary	Baseline vs reduced GIVP-extended model	Maximum/mean ΔNB	0.00048288/0.00009446	–	–	296,950	–
MIMIC-IV internal reduced-vs-full bridge audit	Available-component bridge audit; available reduced score ≥1	Accuracy/Cohen kappa	0.634/0.225	—	—	28,224	—

eICU-CRD was used for reduced external validation because the full three-domain GIVP phenotype could not be reconstructed one-to-one under cross-database variable-availability and time-window constraints. Hospital mortality was used as the available outcome in eICU-CRD. Because infection-source categories could not be harmonized between MIMIC-IV and eICU-CRD with sufficient one-to-one comparability, infection source was not included in the representative eICU adjusted model. APACHE score was used as the primary available severity adjustment variable, and the representative adjusted model was fitted in the model-specific complete-case sample with available APACHE score. The MIMIC-IV bridge audit used an available reduced score ≥ 1 threshold because the MIMIC-IV simulation identified only one eICU-mappable reduced component (mechanical ventilation), constraining the maximum simulated score to 1; this bridge audit threshold is distinct from the component sum ≥ 2 rule applied in the actual eICU-CRD analysis, where multiple EHR-available reduced indicators were available in eICU-CRD. The eICU decision-curve results were interpreted across the evaluated threshold-probability range of 0.05–0.50.

APACHE, acute physiology and chronic health evaluation; APS, acute physiology score; AUC, area under the receiver operating characteristic curve; CI, confidence interval; DCA, decision curve analysis; ΔNB, delta net benefit; EHR, electronic health record; eICU-CRD, eICU collaborative research database; GIVP, gastrointestinal vulnerability phenotype; OR, odds ratio.

**Figure 4:**
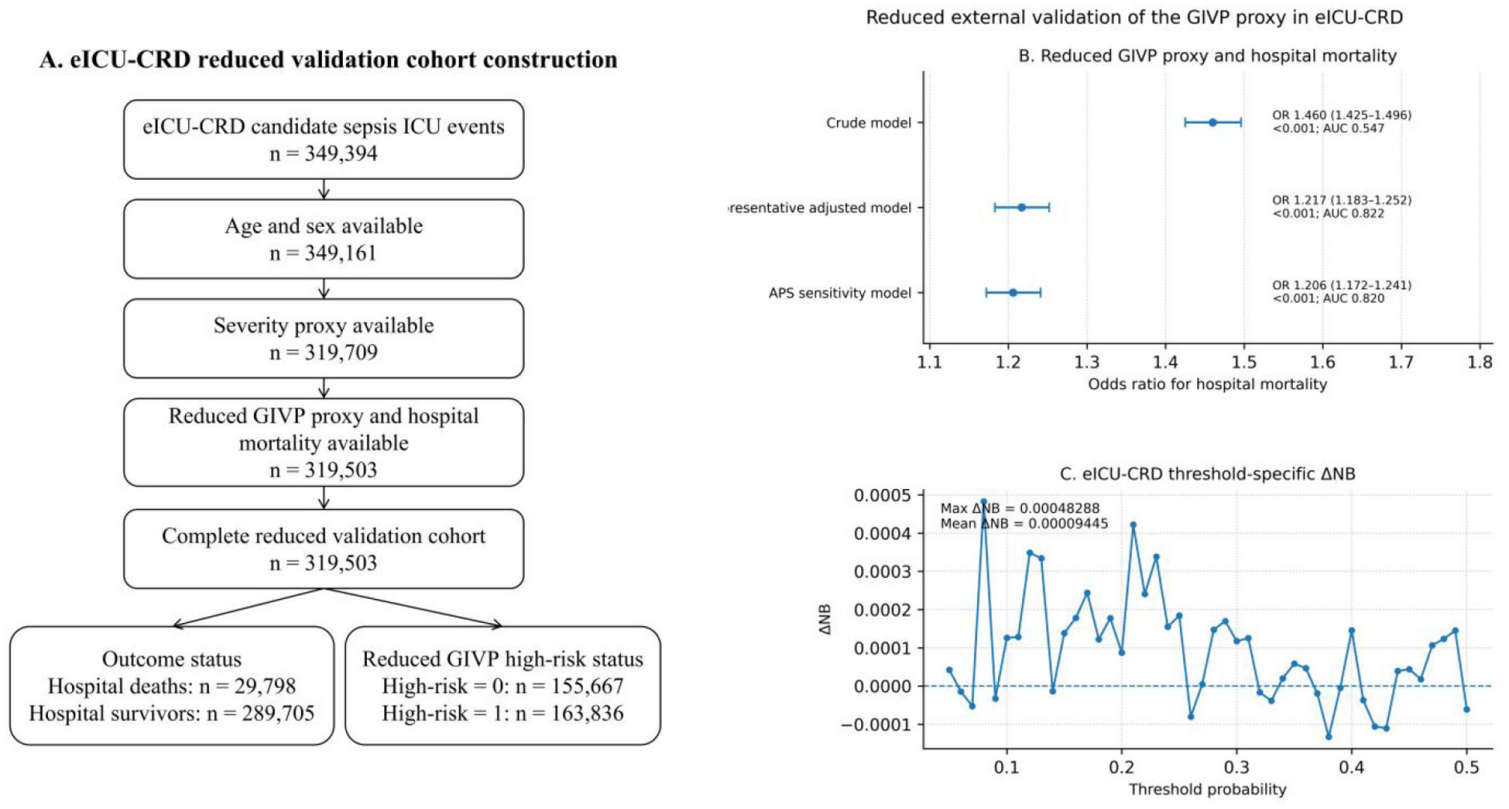
Reduced external validation of the reduced GIVP proxy in eICU-CRD. **(A)** Shows cohort construction and subgroup distributions. **(B)** Shows ORs and 95% CIs for hospital mortality across crude and adjusted models. **(C)** Shows threshold-specific ΔNB across the evaluated threshold-probability range of 0.05–0.50 for the reduced GIVP-extended model vs. the baseline model. This analysis was interpreted as reduced validation rather than exact replication of the full GIVP phenotype. APACHE, Acute Physiology and Chronic Health Evaluation; APS, acute physiology score; CI, confidence interval; ΔNB, delta net benefit; eICU-CRD, eICU Collaborative Research Database; GIVP, gastrointestinal vulnerability phenotype; OR, odds ratio.

The reduced external validation workflow, model estimates, and decision-curve results are visualized in Figure 4.

### 3.7 Cross-level biological interpretability analyses

**Figure 5:**
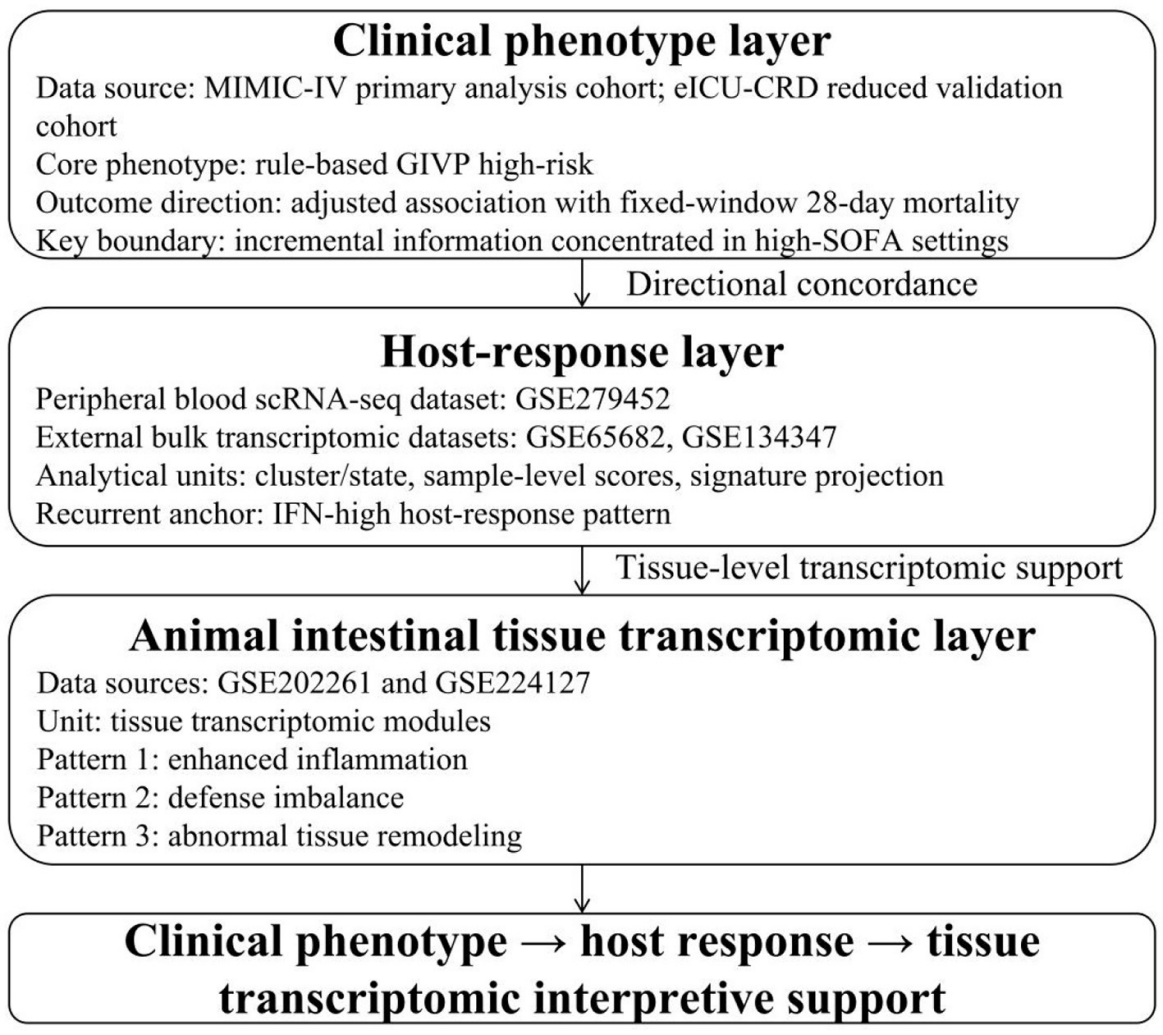
Cross-level interpretive framework for gastrointestinal vulnerability in sepsis. The top, middle, and bottom layers represent the clinical phenotype, host-response, and tissue transcriptomic layers, respectively. The figure illustrates directional concordance among the clinical GIVP phenotype, the IFN-high host-response pattern, and intestinal tissue transcriptomic modules related to inflammation, defense imbalance, and tissue remodeling. This framework was used to support biological plausibility and directional concordance rather than causal inference or patient-level mechanistic inference. GIVP, gastrointestinal vulnerability phenotype; IFN, interferon.

Cross-level biological interpretability analyses were performed to examine whether the clinical GIVP phenotype showed directional concordance with independent host-response and intestinal tissue transcriptomic data layers. These analyses were organized into three linked levels: the clinical phenotype layer, the peripheral host-response layer, and the animal intestinal tissue transcriptomic support layer. Because these datasets were not patient-level matched to the MIMIC-IV cohort, the results were interpreted as biological plausibility and directional concordance rather than causal inference or direct mechanistic inference. The overall cross-level interpretive framework is summarized in Figure 5.

#### 3.7.1 Peripheral host-response support layer

**Figure 6:**
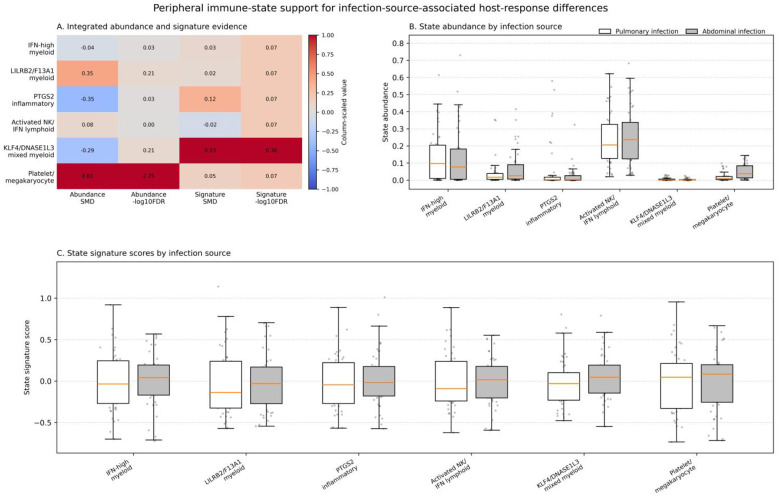
Peripheral host-response support for the IFN-high biological plausibility layer. **(A)** Summarizes integrated state- or cluster-level evidence from the peripheral blood single-cell transcriptomic dataset GSE279452. Rows represent annotated immune states or clusters, and columns summarize abundance-related evidence, statistical evidence for abundance differences, signature-score evidence, statistical evidence for signature-score differences, and integrated support. All displayed evidence metrics were scaled to a ±1 range before visualization. In **(A)** Warm colors indicate stronger support in the abdominal sepsis direction or stronger statistical evidence, whereas cool colors indicate stronger support in the pulmonary sepsis direction or weaker statistical evidence. **(B)** Shows the distribution of immune-state abundance stratified by infection source, comparing abdominal and pulmonary sepsis groups. **(C)** Shows patient-level IFN-high signature scores stratified by infection source, comparing abdominal and pulmonary sepsis groups. In **(B, C)** each point represents an individual sample or patient-level observation as displayed in the plot, boxes indicate the interquartile range, and whiskers summarize the distribution range according to the boxplot convention. Statistical comparisons were performed using the Mann–Whitney *U* test with Benjamini–Hochberg correction where applicable, and effect size was summarized using Cliff's delta. The n values in **(A)** denote the number of immune states or clusters shown, whereas the n values in **(B, C)** denote the number of observations included in each infection-source group. These results were interpreted as host-response-level directional support for biological plausibility and not as patient-level causal inference, matched clinical-omics validation, or direct mechanistic inference for the clinical GIVP phenotype. IFN, interferon; GIVP, gastrointestinal vulnerability phenotype.

The peripheral host-response layer was based on the publicly available peripheral blood mononuclear cell transcriptomic dataset GSE279452 and complemented with signature projection results from the external bulk transcriptomic datasets GSE65682 and GSE134347. Evaluation of this layer relied on cluster/state annotations, sample-level scores, and signature-level results. The IFN-high host-response pattern was used as a recurrent host-response support signal. A summary of the host-response support layer is shown in Figure 6.

#### 3.7.2 Animal intestinal tissue transcriptomic support layer

**Figure 7:**
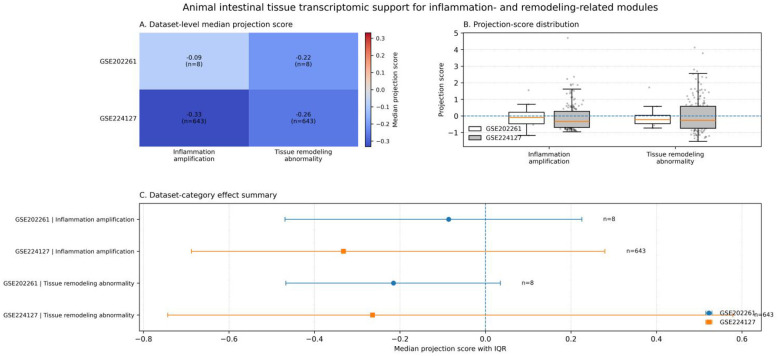
Animal intestinal tissue transcriptomic support for inflammation- and remodeling-related modules. **(A)** Shows dataset-level median projection scores for inflammation amplification and tissue remodeling abnormality modules in two independent animal intestinal tissue transcriptomic datasets: GSE202261, a cecal-ligation-and-puncture model, and GSE224127, a cecal-ligation-and-puncture and lipopolysaccharide time-course model. In **(A)** cell color encodes the median projection score on a symmetric diverging scale, with warm colors indicating positive median scores and cool colors indicating negative median scores. **(B)** Shows the distribution of projection scores stratified by dataset and module category as boxplots; box fill color distinguishes datasets, and individual projection records are overlaid as jittered points. **(C)** Summarizes the median projection score and interquartile range for each dataset–module-category combination; marker shape distinguishes the two datasets. The dashed reference line indicates a projection score of zero. The n values indicate the number of projection records contributing to each summary, rather than the number of animals or biological samples; projection records included raw gene-set scores and *z*-normalized scores; therefore, n does not necessarily equal the number of biological samples. The defense imbalance module category was not represented in the final figure because no mappable projection records were available for that category. Only module categories with mappable projection records were retained. These analyses provide directional tissue-level transcriptomic support for biological plausibility and were not interpreted as causal inference or patient-level mechanistic inference.

The animal intestinal tissue transcriptomic layer was based on two publicly available intestinal tissue transcriptomic datasets included in the module aggregation analysis, GSE202261 and GSE224127. Tissue transcriptomic modules served as the unit of analysis. On the basis of dataset roles and module-level findings, the tissue transcriptomic support layer was summarized into three directionally interpretable module patterns: enhanced inflammation, defense imbalance, and abnormal tissue remodeling. Because the final figure retained only module categories with mappable projection records, Figure 7 displays inflammation amplification and tissue remodeling abnormality modules, whereas the defense imbalance pattern was retained as an interpretive tissue-layer category but was not displayed in the final figure due to the absence of mappable projection records.

#### 3.7.3 Integrated cross-level interpretability

Across the clinical phenotype, host-response, and tissue transcriptomic layers, GIVP high-risk retained an association with fixed-window 28-day mortality after adjustment for clinical context and severity markers, and its additional information was more evident in the high-SOFA setting. The IFN-high host-response pattern and the tissue transcriptomic module patterns summarized as enhanced inflammation, defense imbalance, and abnormal tissue remodeling jointly provided cross-level interpretive support for the biological plausibility of this phenotype. Because these transcriptomic datasets were independent of the clinical cohorts, these findings were treated as cross-level interpretive support rather than patient-level clinical–omics evidence. The host-response and tissue transcriptomic support layers are presented in Figures 6, 7.

## 4 Discussion

In this integrative study, GIVP high-risk was evaluated as a rule-based EHR-operationalized phenotype of early gastrointestinal vulnerability in sepsis. In the MIMIC-IV primary analysis cohort, GIVP high-risk retained an association with fixed-window 28-day mortality after adjustment for clinical context, the continuous SOFA score, and RRT. This association remained directionally consistent in mechanical ventilation-adjusted analysis, unit-of-analysis sensitivity analyses, exchangeable-correlation GEE, repeated random single-record selection, and supportive Cox sensitivity analysis. Incremental prediction and DCA showed only small additional risk-stratification information overall, with the signal more concentrated in the high-SOFA subgroup. Reduced validation in eICU-CRD supported external prognostic directionality, whereas cross-level transcriptomic analyses supported biological plausibility, without establishing exact external replication or causal inference (32–34). GIVP should be interpreted in relation to existing bedside gastrointestinal assessment systems, but not as their replacement. The AGI grading framework provides a clinically oriented approach to gastrointestinal dysfunction in critical illness, but its application depends on bedside findings such as feeding intolerance, abdominal distension, gastric residuals, bowel sounds, intra-abdominal pressure, and clinician-integrated assessment. These variables are often incompletely captured, inconsistently coded, or unavailable as structured fields in large ICU databases. In contrast, GIVP was designed as an EHR-operationalized phenotype that uses reproducible early ICU variables to summarize gastrointestinal vulnerability within database constraints. Therefore, its intended role is complementary: it provides a scalable analytical phenotype for retrospective and multi-database research, rather than a bedside substitute for AGI grading or direct gastrointestinal function assessment.

A key finding was that the prognostic relevance of GIVP high-risk was not reducible to infection source alone. Infection source is closely related to organ involvement, supportive care trajectories, and outcome heterogeneity in sepsis. In the present analysis, GIVP high-risk retained an association with fixed-window 28-day mortality after adjustment for age, sex, Charlson Comorbidity Index, infection source, the continuous SOFA score, and RRT. However, this finding should not be interpreted as complete separation from acute severity. The lactate-overadjusted model yielded a directionally reversed estimate, indicating structural overlap between GIVP and proximal metabolic severity, particularly because lactate contributed to the hypoperfusion-related component of the phenotype. Therefore, lactate adjustment was interpreted as an overadjustment sensitivity analysis rather than as evidence that GIVP high-risk had a protective effect. These findings suggest that GIVP may capture a gastrointestinal vulnerability signal that is not reducible to infection source alone, while still partly overlapping with acute severity burden (35–38).

The incremental prediction results help clarify the appropriate clinical interpretation of GIVP high-risk. In the full cohort, the increase in AUC after adding GIVP high-risk was very small, and DCA showed only a small net-benefit gain. In the high-SOFA subgroup, the incremental signal was more apparent, but the absolute improvement remained modest. Because no universally accepted single decision threshold exists for applying an EHR-derived gastrointestinal vulnerability phenotype to guide sepsis management, the DCA findings should be interpreted as exploratory evidence of limited supplementary clinical utility rather than as evidence that GIVP high-risk can directly guide clinical decision-making. These findings support positioning GIVP high-risk as a complementary risk-stratification phenotype, particularly among patients with higher global severity, rather than as a standalone prediction tool or substitute for established severity scores (37, 38). The supportive analyses using GIVP score and AB sum were directionally consistent with this interpretation, suggesting that the signal was not confined to one dichotomous threshold alone (39–44). The eICU analysis should also be interpreted within the boundary of reduced validation. The full three-domain GIVP phenotype could not be reconstructed one-to-one in eICU-CRD because of cross-database differences in variable availability, time-window definitions, infection-source harmonization, and outcome structure. In addition, the nutrition-related reduced indicators had limited discriminative contribution in eICU-CRD because absence of enteral nutrition initiation within 24 h was present in nearly all ICU admission events, whereas delayed enteral nutrition initiation beyond 24 h contributed no positive events. Therefore, the eICU association mainly reflected the external prognostic directionality of an EHR-available reduced proxy driven by mechanical ventilation and sedation-related variation on the background of near-universal early enteral nutrition absence, rather than exact transportability of the full nutritional-implementation domain. The MIMIC-IV internal bridge audit, which used an available reduced score ≥1 threshold because only one eICU-mappable reduced component was identified in the MIMIC-IV simulation, showed only partial agreement between the reduced GIVP proxy and the full GIVP construct (accuracy 0.634, Cohen kappa 0.225). In the actual eICU-CRD analysis, the reduced GIVP proxy was defined using a component sum ≥2 threshold because multiple EHR-available reduced indicators were available. Thus, reduced GIVP high-risk remained directionally associated with hospital mortality after adjustment for age, sex, and APACHE score in eICU-CRD, but this finding supports conceptual prognostic transportability rather than full external replication of the MIMIC-IV three-domain GIVP phenotype.

The cross-level analyses provided interpretive biological support rather than direct mechanistic inference. The IFN-high host-response pattern and the intestinal tissue module patterns, summarized as enhanced inflammation, defense imbalance, and abnormal tissue remodeling, were compatible with biological themes relevant to sepsis. Because these transcriptomic datasets were independent of the clinical cohorts and were not linked at the patient level, they could not establish mediation between GIVP high-risk and mortality (32–36, 45).

Several limitations should be acknowledged. First, this was a retrospective database-based study, and the observed associations should not be interpreted as causal effects. Although unit-of-analysis sensitivity analyses, including first-ICU restriction, subject-level robust inference, exchangeable-correlation GEE, and repeated random single-record selection, supported the robustness of the primary association, residual dependence and unmeasured confounding cannot be fully excluded. Second, GIVP high-risk is an EHR-operationalized phenotype rather than a standardized bedside measure of gastrointestinal dysfunction. It does not replace AGI grading, direct feeding tolerance assessment, intra-abdominal pressure measurement, or clinician-adjudicated gastrointestinal evaluation. Third, although Cox sensitivity analysis showed directional consistency, the Cox sensitivity dataset was reconstructed separately according to survival-time availability and was not identical to the complete-case fixed-window logistic primary analysis cohort. Therefore, the Cox analysis was used only to examine whether the association was directionally consistent under a time-to-event framework and was not used to replace, recalibrate, or supersede the primary fixed-window logistic inference. In addition, proportional hazards diagnostics and time-stratified event patterns suggested possible non-proportionality; therefore, Cox regression was interpreted as supportive, and fixed-window logistic regression remained the primary inferential model. Fine-Gray competing-risk modeling was not pursued because competing discharge and censoring structures could not be standardized across the reconstructed analytic datasets. Fourth, the lactate-overadjusted model showed a directionally reversed estimate, supporting the interpretation that lactate overlaps with the hypoperfusion component of GIVP and proximal metabolic severity; this limits claims regarding severity-independent effects. Fifth, the incremental prediction and decision-curve gains were small, particularly in the full cohort, indicating that GIVP high-risk should be viewed as a complementary phenotype rather than a clinically decisive prediction model. Sixth, the eICU analysis used a reduced GIVP proxy defined by a component sum ≥2 threshold and hospital mortality as the outcome, and therefore supported directional external transportability rather than exact replication of the full MIMIC-IV GIVP phenotype based on 28-day mortality. The nutrition-related reduced indicators were also structurally imbalanced in eICU-CRD: absence of enteral nutrition initiation within 24 h was present in nearly all ICU admission events, whereas delayed enteral nutrition initiation beyond 24 h contributed no positive events. The eICU reduced proxy therefore mainly reflected mechanical ventilation and sedation-related variation superimposed on a near-universal early enteral nutrition absence signal. In addition, the MIMIC-IV internal bridge audit used an available reduced score ≥1 threshold reflecting the single eICU-mappable component available in the MIMIC-IV simulation and showed partial agreement (accuracy 0.634, Cohen kappa 0.225), further indicating that the reduced proxy should not be interpreted as an exact substitute for the full three-domain GIVP phenotype. Finally, the host-response and animal intestinal tissue transcriptomic datasets were independent of the clinical cohorts; thus, the cross-level findings support biological plausibility but not patient-level matched clinical–omics evidence or causal inference. Prospective studies with standardized gastrointestinal phenotyping, complete time-to-event outcome capture, and patient-level multi-omics measurements are needed (33, 34, 37–40, 45).

## 5 Conclusion

GIVP high-risk is a prespecified, rule-based EHR-operationalized phenotype that captures early gastrointestinal vulnerability in sepsis. In the MIMIC-IV primary analysis cohort, it retained an adjusted prognostic association with fixed-window 28-day mortality after adjustment for clinical context, the continuous SOFA score, and RRT, and the association remained directionally consistent across multiple sensitivity analyses. Its incremental prediction and decision-curve gains were small overall, with the signal being more concentrated in the high-SOFA subgroup. Reduced external validation in eICU-CRD supported external prognostic directionality, whereas cross-level transcriptomic analyses supported biological plausibility, but not exact external replication or causal inference. Together, these findings position GIVP high-risk as a complementary EHR-based gastrointestinal vulnerability phenotype for sepsis risk stratification, rather than as a replacement for established severity scores or bedside gastrointestinal assessment systems.

## Data Availability

This study analyzed publicly available datasets from MIMIC-IV, eICU-CRD, and GEO datasets GSE279452, GSE65682, GSE134347, GSE202261, and GSE224127. The derived results supporting the conclusions of this article are included in the article and Supplementary material. Further inquiries can be directed to the corresponding author.

## References

[1] Singer M Deutschman CS Seymour CW Shankar-Hari M Annane D Bauer M . The third international consensus definitions for sepsis and septic shock (Sepsis-3). JAMA. (2016) 315:801–10. doi: 10.1001/jama.2016.0287 26903338 PMC4968574

[2] Seymour CW Kennedy JN Wang S Chang CH Elliott CF Xu Z . Derivation, validation, and potential treatment implications of novel clinical phenotypes for sepsis. JAMA. (2019) 321:2003–17. doi: 10.1001/jama.2019.5791 31104070 PMC6537818

[3] Reintam Blaser A Malbrain MLNG Starkopf J Fruhwald S Jakob SM De Waele J . Gastrointestinal function in intensive care patients: terminology, definitions and management. Recommendations of the ESICM working group on abdominal problems. Intensive Care Med. (2012) 38:384–94. doi: 10.1007/s00134-011-2459-y 22310869 PMC3286505

[4] Reintam Blaser A Preiser JC Fruhwald S Wilmer A Wernerman J Benstoem C . Gastrointestinal dysfunction in the critically ill: a systematic scoping review and research agenda proposed by the Section of Metabolism, Endocrinology and Nutrition of the European Society of Intensive Care Medicine. Crit Care. (2020) 24:224. doi: 10.1186/s13054-020-02889-4 32414423 PMC7226709

[5] McClave SA Gualdoni J Nagengast A Marsano LS Bandy K Martindale RG. Gastrointestinal dysfunction and feeding intolerance in critical illness: do we need an objective scoring system? Curr Gastroenterol Rep. (2020) 22:1. doi: 10.1007/s11894-019-0736-z 31912312

[6] Zhang D Li Y Ding L Fu Y Dong X Li H. Prevalence and outcome of acute gastrointestinal injury in critically ill patients: a systematic review and meta-analysis. Medicine. (2018) 97:e12970. doi: 10.1097/MD.0000000000012970 30412121 PMC6221717

[7] Li H Zhang D Wang Y Zhao S. Association between acute gastrointestinal injury grading system and disease severity and prognosis in critically ill patients: a multicenter, prospective, observational study in China. J Crit Care. (2016) 36:24–8. doi: 10.1016/j.jcrc.2016.05.001 27546743

[8] Peters-Sengers H Butler JM Uhel F Schultz MJ Bonten MJM Cremer OL . Source-specific host response and outcomes in critically ill patients with sepsis: a prospective cohort study. Intensive Care Med. (2022) 48:92–102. doi: 10.1007/s00134-021-06574-0 34902047 PMC8667541

[9] Assimakopoulos SF Triantos C Thomopoulos K Fligou F Maroulis I Marangos M . Gut-origin sepsis in the critically ill patient: pathophysiology and treatment. Infection. (2018) 46:751–60. doi: 10.1007/s15010-018-1178-5 30003491

[10] Johnson AEW Bulgarelli L Shen L Gayles A Shammout A Horng S . MIMIC-IV, a freely accessible electronic health record dataset. Sci Data. (2023) 10:1. doi: 10.1038/s41597-023-01945-2 36596836 PMC9810617

[11] Pollard TJ Johnson AEW Raffa JD Celi LA Mark RG Badawi O. The eICU Collaborative Research Database, a freely available multi-center database for critical care research. Sci Data. (2018) 5:180178. doi: 10.1038/sdata.2018.178 30204154 PMC6132188

[12] Collins GS Reitsma JB Altman DG Moons KGM. Transparent reporting of a multivariable prediction model for individual prognosis or diagnosis (TRIPOD): the TRIPOD statement. Ann Intern Med. (2015) 162:55–63. doi: 10.7326/M14-0697 25560714

[13] Moons KGM Altman DG Reitsma JB Ioannidis JPA Macaskill P Steyerberg EW . Transparent reporting of a multivariable prediction model for individual prognosis or diagnosis (TRIPOD): explanation and elaboration. Ann Intern Med. (2015) 162:W1–W73. doi: 10.7326/M14-0698 25560730

[14] von Elm E Altman DG Egger M Pocock SJ Gøtzsche PC Vandenbroucke JP STROBE Initiative. The strengthening the reporting of observational studies in epidemiology (STROBE) statement: guidelines for reporting observational studies. Ann Intern Med. (2007) 147:573–7. doi: 10.7326/0003-4819-147-8-200710160-00010 17938396

[15] Vandenbroucke JP von Elm E Altman DG Gøtzsche PC Mulrow CD Pocock SJ . Strengthening the reporting of observational studies in epidemiology (STROBE): explanation and elaboration. Epidemiology. (2007) 18:805–35. doi: 10.1097/EDE.0b013e3181577511 18049195

[16] Steyerberg EW Vickers AJ Cook NR Gerds T Gonen M Obuchowski N . Assessing the performance of prediction models: a framework for traditional and novel measures. Epidemiology. (2010) 21:128–38. doi: 10.1097/EDE.0b013e3181c30fb2 20010215 PMC3575184

[17] Edgar R Domrachev M Lash AE. Gene expression omnibus: NCBI gene expression and hybridization array data repository. Nucleic Acids Res. (2002) 30:207–10. doi: 10.1093/nar/30.1.207 11752295 PMC99122

[18] Davis S Meltzer PS. GEOquery: a bridge between the Gene Expression Omnibus (GEO) and BioConductor. Bioinformatics. (2007) 23:1846–7. doi: 10.1093/bioinformatics/btm254 17496320

[19] Wolf FA Angerer P Theis FJ. SCANPY: large-scale single-cell gene expression data analysis. Genome Biol. (2018) 19:15. doi: 10.1186/s13059-017-1382-0 29409532 PMC5802054

[20] Davenport EE Burnham KL Radhakrishnan J Humburg P Hutton P Mills TC . Genomic landscape of the individual host response and outcomes in sepsis: a prospective cohort study. Lancet Respir Med. (2016) 4:259–71. doi: 10.1016/S2213-2600(16)00046-1 26917434 PMC4820667

[21] Scicluna BP Uhel F van Vught LA Wiewel MA Hoogendijk AJ Baessman I . The leukocyte non-coding RNA landscape in critically ill patients with sepsis. Elife. (2020) 9:e58597. doi: 10.7554/eLife.58597 33305733 PMC7775110

[22] Sweeney TE Azad TD Donato M Haynes WA Perumal TM Henao R . Unsupervised analysis of transcriptomics in bacterial sepsis across multiple datasets reveals three robust clusters. Crit Care Med. (2018) 46:915–25. doi: 10.1097/CCM.0000000000003084 29537985 PMC5953807

[23] Hong Y Chen L Sun J Xing L Yang Y Jin X . Single-cell transcriptome profiling reveals heterogeneous neutrophils with prognostic values in sepsis. iScience. (2022) 25:105301. doi: 10.1016/j.isci.2022.105301 36304125 PMC9593767

[24] Vincent JL Moreno R Takala J Willatts S De Mendonca A Bruining H . The SOFA (Sepsis-related Organ Failure Assessment) score to describe organ dysfunction/failure. Intensive Care Med. (1996) 22:707–10. doi: 10.1007/BF01709751 8844239

[25] Charlson ME Pompei P Ales KL MacKenzie CR. A new method of classifying prognostic comorbidity in longitudinal studies: development and validation. J Chronic Dis. (1987) 40:373–83. doi: 10.1016/0021-9681(87)90171-8 3558716

[26] DeLong ER DeLong DM Clarke-Pearson DL. Comparing the areas under two or more correlated receiver operating characteristic curves: a nonparametric approach. Biometrics. (1988) 44:837–45. doi: 10.2307/2531595 3203132

[27] Demler OV Pencina MJ D'Agostino RB Sr. Misuse of DeLong test to compare AUCs for nested models. Stat Med. (2012) 31:2577–87. doi: 10.1002/sim.5328 22415937 PMC3684152

[28] Brier GW. Verification of forecasts expressed in terms of probability. Mon Weather Rev. (1950) 78:1–3. doi: 10.1175/1520-0493(1950)078 < 0001:VOFEIT>2.0.CO;2

[29] Van Calster B Nieboer D Vergouwe Y De Cock B Pencina MJ Steyerberg EW . Calibration hierarchy for risk models was defined: from utopia to empirical data. J Clin Epidemiol. (2016) 74:167–76. doi: 10.1016/j.jclinepi.2015.12.005 26772608

[30] Van Calster B McLernon DJ van Smeden M Wynants L Steyerberg EW Topic Topic Group Evaluating Diagnostic Tests and Prediction Models of the STRATOS Initiative. Calibration: the achilles heel of predictive analytic. BMC Med. (2019) 17:230. doi: 10.1186/s12916-019-1466-7 31842878 PMC6912996

[31] Pencina MJ D'Agostino RB Sr Steyerberg EW. Extensions of net reclassification improvement calculations to measure usefulness of new biomarkers. Stat Med. (2011) 30:11–21. doi: 10.1002/sim.4085 21204120 PMC3341973

[32] Agudelo-Ochoa GM Valdés-Duque BE Giraldo-Giraldo NA Jaillier-Ramírez AM Giraldo-Villa A Acevedo-Castaño I . Gut microbiota profiles in critically ill patients, potential biomarkers and risk variables for sepsis. Gut Microbes. (2020) 12:1707610. doi: 10.1080/19490976.2019.1707610 31924126 PMC7524144

[33] Shimizu K Ogura H Oda J. Gut dysbiosis and its treatment in patients with critical illness. Acute Med Surg. (2025) 12:e70068. doi: 10.1002/ams2.70068 40469413 PMC12133608

[34] Piccioni A Spagnuolo F Candelli M Voza A Covino M Gasbarrini A . The gut microbiome in sepsis: from dysbiosis to personalized therapy. J Clin Med. (2024) 13:6082. doi: 10.3390/jcm13206082 39458032 PMC11508704

[35] Nieuwenhuijzen GA Goris RJ. The gut: the motor of multiple organ dysfunction syndrome? Curr Opin Clin Nutr Metab Care. (1999) 2:399–404. doi: 10.1097/00075197-199909000-00008 10589382

[36] Rotstein OD. Pathogenesis of multiple organ dysfunction syndrome: gut origin, protection, and decontamination. Surg Infect. (2000) 1:217–25. doi: 10.1089/109629600750018141 12594892

[37] Shen C Wang X Xiao YY Zhang JY Xia GL Jiang RL. Comparing gastrointestinal dysfunction score and acute gastrointestinal injury grade for predicting short-term mortality in critically ill patients. World J Gastroenterol. (2024) 30:4523–31. doi: 10.3748/wjg.v30.i42.4523 39563745 PMC11572625

[38] Hai PD Tot NH Thao LT Khoa Q Thien DH. Prognostic value of acute gastrointestinal injury combined with disease severity scores in critically ill patients. Indian J Crit Care Med. (2024) 28:575–80. doi: 10.5005/jp-journals-10071-24733 39130390 PMC11310679

[39] Singer P Blaser AR Berger MM Alhazzani W Calder PC Casaer MP . ESPEN guideline on clinical nutrition in the intensive care unit. Clin Nutr. (2019) 38:48–79. doi: 10.1016/j.clnu.2018.08.037 30348463

[40] Reintam Blaser A Starkopf J Alhazzani W Berger MM Casaer MP Deane AM . Early enteral nutrition in critically ill patients: ESICM clinical practice guidelines. Intensive Care Med. (2017) 43:380–98. doi: 10.1007/s00134-016-4665-0 28168570 PMC5323492

[41] Jiang Y Hu B Zhang S Cai M Chu X Zheng D . Effects of early enteral nutrition on the prognosis of patients with sepsis: secondary analysis of acute gastrointestinal injury study. Ann Palliat Med. (2020) 9:3793–801. doi: 10.21037/apm-20-1650 33302649

[42] Sun JK Nie S Chen YM Zhou J Wang X Zhou SM . Effects of permissive hypocaloric vs standard enteral feeding on gastrointestinal function and outcomes in sepsis. World J Gastroenterol. (2021) 27:4900–12. doi: 10.3748/wjg.v27.i29.4900 34447234 PMC8371509

[43] Moon SJ Ko RE Park CM Suh GY Hwang J Chung CR. The effectiveness of early enteral nutrition on clinical outcomes in critically ill sepsis patients: a systematic review. Nutrients. (2023) 15:3201. doi: 10.3390/nu15143201 37513620 PMC10383540

[44] Xu W Zhong M Pan T Qu H Chen E. Gut microbiota and enteral nutrition tolerance in non-abdominal infection septic ICU patients: an observational study. Nutrients. (2022) 14:5342. doi: 10.3390/nu14245342 36558501 PMC9783285

[45] Liu L Yue Q Chen J Liu H Zeng X. Intestinal injury signaling pathway in sepsis. Front Immunol. (2025) 16:1620965. doi: 10.3389/fimmu.2025.1620965 40655155 PMC12245687

