# Supplementary material for: The high-risk phenotype for gastrointestinal vulnerability in sepsis and 28-day mortality: an integrative study based on clinical association and cross-level biological support
Source: Front Med (Lausanne). 2026 Jun 30;13:1854067. doi: 10.3389/fmed.2026.1854067 (PMC13365328; doi:10.3389/fmed.2026.1854067)
Supplement: Supplementary file 1 [file Supplementary_file_1.docx]

**Supplementary Tables for GIVP-Derived Analyses**

**Supplementary Table S1. Full-to-reduced GIVP mapping and internal bridge audit for the eICU-available reduced proxy**

| Bridge analysis | Threshold definition | Sample size | Reduced high-risk, n | Confusion matrix, TP/FP/FN/TN | Accuracy | Diagnostic performance, Se/Sp/PPV/NPV/BalAcc | Cohen kappa | Interpretation |
| --- | --- | --- | --- | --- | --- | --- | --- | --- |
| Available-component informative bridge audit | Available reduced score ≥1 | 28,224 | 16,468 | 12,551 / 3,917 / 6,400 / 5,356 | 0.634 | 0.662 / 0.578 / 0.762 / 0.456 / 0.620 | 0.225 | Partial agreement; supports reduced external directional consistency, not exact full-phenotype replication |
| Degenerate threshold check | Reduced score ≥2 | 28,224 | 0 | 0 / 0 / 18,951 / 9,273 | 0.329 | 0.000 / 1.000 / NA / 0.329 / 0.500 | 0.000 | Degenerate threshold in the MIMIC-IV bridge simulation because the simulated reduced score ranged only from 0 to 1; not used for the informative bridge comparison |

*Note: The full reference was full MIMIC-IV GIVP high-risk, defined using the three-domain rule-based EHR-operationalized GIVP phenotype. The reduced proxy was the MIMIC-IV simulated reduced GIVP based on eICU-mappable components. In the MIMIC-IV bridge dataset, only one eICU-mappable reduced component, mechanical ventilation, could be reconstructed; therefore, the simulated reduced score ranged from 0 to 1, and the informative bridge comparison used available reduced score ≥1. Using a ≥2 threshold in this MIMIC-IV bridge simulation classified no patients as reduced high-risk and yielded sensitivity = 0 and Cohen kappa = 0, with PPV not estimable because there were no predicted positives; this degenerate threshold was therefore not used for the informative bridge comparison. In the actual eICU-CRD analysis, multiple EHR-available reduced indicators were available, and reduced GIVP high-risk was defined using a component sum ≥2 threshold. These two thresholds reflect different implementation contexts, namely MIMIC-IV bridge simulation versus eICU-CRD actual analysis, and should not be interpreted as conflicting definitions. TP, true positive; FP, false positive; FN, false negative; TN, true negative; Se, sensitivity; Sp, specificity; PPV, positive predictive value; NPV, negative predictive value; BalAcc, balanced accuracy; EHR, electronic health record; eICU-CRD, eICU Collaborative Research Database; GIVP, gastrointestinal vulnerability phenotype; MIMIC-IV, Medical Information Mart for Intensive Care IV.*

**Supplementary Table S2. Formula validation for GIVP-derived variables**

| **Construct** | **Validation rule** | **n_evaluable** | **Mismatch** | **Accuracy** | **Validation status** |
| --- | --- | --- | --- | --- | --- |
| Domain A | domain_a_main_flag = vaso_any_24h == 1 OR lactate_max_24h ≥ 4 | 41295 | 0 | 1 | Exact |
| Domain B | domain_b_main_flag_derived = domain_b_derived | 28224 | 0 | 1 | Exact at final analysis-variable level |
| Domain C | domain_c_main_flag = fluid_path_flag == 1 OR imv_sed_path_flag == 1 | 41295 | 0 | 1 | Exact |
| GIVP score | GIVP score = Domain A + Domain B + Domain C | 28224 | 0 | 1 | Exact |
| GIVP high-risk | GIVP high-risk = GIVP score ≥ 2 | 28224 | 0 | 1 | Exact |
| AB sum | AB sum = Domain A + Domain B | 28224 | 0 | 1 | Exact |

*Note: Formula validation was used to confirm that the derived GIVP score, GIVP high-risk status, and AB sum could be exactly reconstructed from their prespecified component domains.Component-level validation for Domain A and Domain C was performed in the broader domain-reconstructable candidate dataset, whereas GIVP score, GIVP high-risk, AB sum, and Domain B validation were evaluated in the final primary analysis dataset.*

*Abbreviations: GIVP, gastrointestinal vulnerability phenotype.*

****Supplementary Table S3. Evidence-based operational source definitions for GIVP component domains****

| GIVP domain | Evidence source in the analysis workflow | Analysis variable or derived flag | Operational criterion confirmed from files | Time window confirmed from files | Coding rule confirmed from files | Notes on reproducibility and interpretation |
| --- | --- | --- | --- | --- | --- | --- |
| Domain A: vasoactive-agent exposure | S01_GIVP_operational_definition.md; Supplementary_Table_formula_validation.csv; A034_validate_final_domain_definitions.py | vaso_any_24h | Vasoactive-agent exposure contributes to Domain A when vaso_any_24h == 1. | 0–24 h after ICU admission | Confirmed as one arm of Domain A: domain_a_main_flag = 1 if vaso_any_24h == 1 or lactate_max_24h ≥4 mmol/L; otherwise 0. | Underlying itemid-level vasopressor mapping was not itemized in the scanned final definition tables; this row therefore reports the validated analysis-level exposure flag rather than an item-level medication list. |
| Domain A: lactate maximum | S01_GIVP_operational_definition.md; Supplementary_Table_formula_validation.csv; A034_validate_final_domain_definitions.py | lactate_max_24h | Lactate contributes to Domain A when maximum lactate is ≥4 mmol/L. | 0–24 h after ICU admission | Confirmed as one arm of Domain A: domain_a_main_flag = 1 if vaso_any_24h == 1 or lactate_max_24h ≥4 mmol/L; otherwise 0. | Threshold confirmed from scanned files; no new lactate threshold introduced. |
| Domain A: final domain flag | S01_GIVP_operational_definition.md; Supplementary_Table_formula_validation.csv; A034_validate_final_domain_definitions.py | domain_a_main_flag | Early hemodynamic support or hypoperfusion. | 0–24 h after ICU admission | Confirmed exact: domain_a_main_flag = 1 if vaso_any_24h == 1 or lactate_max_24h ≥4 mmol/L; otherwise 0. Formula validation showed mismatch 0 and accuracy 1.000. | Reproducible from final analysis variables. |
| Domain B: enteral nutrition initiation | S01_GIVP_operational_definition.md; Supplementary_Table_formula_validation.csv; A034_validate_final_domain_definitions.py; 02_python/52_build_givp_trajectory_dataset_v1.py | domain_b_main_flag_derived; domain_b_derived; EN timing variables referenced as first_en_time / en_any_72h | Absence of qualifying enteral nutrition initiation; final validation confirms domain_b_main_flag_derived = domain_b_derived. | 0–72 h after ICU admission | Confirmed exact at final analysis-variable level; EN source logic referenced through en_any_72h / first_en_time, but final validated rule is derived-flag equivalence. | Final Domain B reconstruction was validated at the analysis-variable level. EN timing variables were identified in scanned scripts, but bottom-level EN itemid mapping was not identified in the scanned final supplementary tables or result files. |
| Domain C: fluid exposure pathway | S01_GIVP_operational_definition.md; Supplementary_Table_formula_validation.csv; A034_validate_final_domain_definitions.py; 02_python/09_build_domain_c.py | fluid_path_flag; fluid_total_ml_24h | Total qualifying fluid volume ≥2,000 mL contributes to Domain C. | 0–24 h after ICU admission | Confirmed in script as fluid_path_flag = 1 when fluid_total_ml_24h was ≥2,000 mL; also confirmed as one arm of the Domain C formula. | Fluid itemid values not itemized in final S1; script contains rule-based fluid item selection logic. |
| Domain C: invasive mechanical ventilation with sedative/opioid exposure | S01_GIVP_operational_definition.md; Supplementary_Table_formula_validation.csv; A034_validate_final_domain_definitions.py; 02_python/09_build_domain_c.py | imv_sed_path_flag; first_imv_time; first_sed_time | Invasive mechanical ventilation combined with sedative/opioid exposure contributes to Domain C. | 0–24 h after ICU admission | Confirmed in script as imv_sed_path_flag = 1 when both first_imv_time and first_sed_time were non-null within the 24 h derivation window; also confirmed as one arm of the Domain C formula. | Sedative/opioid label-selection logic was identified in the scanned script; exact itemid-level medication lists were not itemized in the scanned final supplementary tables. |
| Domain C: final domain flag | S01_GIVP_operational_definition.md; Supplementary_Table_formula_validation.csv; A034_validate_final_domain_definitions.py; 02_python/09_build_domain_c.py | domain_c_main_flag | Iatrogenic exposure pathway. | 0–24 h after ICU admission | Confirmed exact: domain_c_main_flag = 1 if fluid_path_flag == 1 or imv_sed_path_flag == 1; otherwise 0. Formula validation showed mismatch 0 and accuracy 1.000. | Reproducible from final pathway flags. |
| GIVP score | S01_GIVP_operational_definition.md; S02_GIVP_formula_validation.csv; Supplementary_Table_formula_validation.csv; A018_compile_final_revision_evidence_package.py | givp_score_main_v1 | Composite of Domain A + Domain B + Domain C. | Composite early ICU window | Confirmed exact: givp_score_main_v1 = domain_a_main_flag + domain_b_main_flag_derived + domain_c_main_flag; mismatch 0, accuracy 1.000. | Rule-based score from final binary domain flags. |
| GIVP high-risk | S01_GIVP_operational_definition.md; S02_GIVP_formula_validation.csv; Supplementary_Table_formula_validation.csv; A009_make_givp_definition_tables.py; A018_compile_final_revision_evidence_package.py | givp_highrisk_main_v1 | High-risk phenotype defined by GIVP score threshold. | Composite early ICU window | Confirmed exact: givp_highrisk_main_v1 = 1 if givp_score_main_v1 ≥2; otherwise 0. Formula validation showed mismatch 0 and accuracy 1.000. | Deterministic threshold phenotype, not an outcome-trained model. |
| AB sum | S01_GIVP_operational_definition.md; S02_GIVP_formula_validation.csv; Supplementary_Table_formula_validation.csv; 05_run_logistic_givp_ab_completecase_vs_domainbeligible_v1.py; A018_compile_final_revision_evidence_package.py | ab_sum_v1 | Sum of Domain A and Domain B. | Domain A 0–24 h after ICU admission; Domain B 0–72 h after ICU admission | Confirmed exact: ab_sum_v1 = domain_a_main_flag + domain_b_main_flag_derived; mismatch 0, accuracy 1.000. | Supportive cumulative-burden construct; not identical to GIVP high-risk. |

*Note: This table was generated from the internal analysis workflow, validated formula tables, result files, and supplementary tables used for the revised analysis. It does not introduce new variables, thresholds, or analyses. It summarizes the evidence-supported analysis variables, derived flags, time windows, and coding rules used for GIVP construction. Variables or detailed item-level mappings not identified in the available analysis materials are explicitly marked as not itemized rather than inferred.*

*Abbreviations: AB sum, sum of Domain A and Domain B; AGI, acute gastrointestinal injury; EHR, electronic health record; EN, enteral nutrition; GIVP, gastrointestinal vulnerability phenotype; ICU, intensive care unit; IMV, invasive mechanical ventilation.*

**Supplementary Table S4. Interpretation boundaries for reduced external validation and cross-level biological analyses**

| **Layer** | **Data source** | **Analytical unit** | **Main finding type** | **Permitted interpretation** | **Interpretation boundary** |
| --- | --- | --- | --- | --- | --- |
| Clinical phenotype layer | MIMIC-IV primary clinical analysis cohort | ICU admission event | Association with fixed-window 28-day mortality | Prognostic association after adjustment for clinical context and severity-related variables | Retrospective association; not causal inference |
| Reduced external validation layer | eICU-CRD | ICU admission event | Reduced proxy association with hospital mortality | Directional external prognostic transportability of an EHR-available reduced proxy | Not exact external replication of full three-domain GIVP |
| Peripheral host-response layer | Public peripheral blood single-cell and bulk transcriptomic datasets | Host-response state or signature-level result | IFN-high host-response support signal | Biological plausibility and directional concordance | Not patient-level closed-loop validation; not causal mechanism proof |
| Animal intestinal tissue transcriptomic layer | Public animal intestinal tissue transcriptomic datasets | Tissue transcriptomic module | Enhanced inflammation, defense imbalance, abnormal tissue remodeling | Cross-level interpretive support | Independent animal data; not direct mediation analysis in clinical patients |

*Note: Cross-level biological analyses were designed to support biological plausibility and directional concordance. They were not used to establish patient-level causal inference, mechanistic inference, or direct mediation between the clinical phenotype and mortality.*

*Abbreviations: GIVP, gastrointestinal vulnerability phenotype.*

**Supplementary Table S5. Component distribution according to GIVP high-risk status**

| **GIVP high-risk status** | **n** | **28-day deaths, n**  **(%)** | **Domain A positive, n**  **(%)** | **Domain B positive, n**  **(%)** | **Domain C positive, n**  **(%)** | **Mean GIVP score** |
| --- | --- | --- | --- | --- | --- | --- |
| Low-risk | 9273 | 2,172 (23.42%) | 1,124 (12.12%) | 6,038 (65.11%) | 1,135 (12.24%) | 0.8947 |
| High-risk | 18951 | 4,690 (24.75%) | 15,301 (80.74%) | 16,331 (86.17%) | 15,519 (81.89%) | 2.488 |

*Note: This table describes the accumulation pattern of Domain A, Domain B, and Domain C across GIVP high-risk strata. The results support the interpretation of GIVP high-risk as a multi-domain early vulnerability phenotype rather than a single-variable marker.*

*Abbreviations: GIVP, gastrointestinal vulnerability phenotype.*

**Supplementary Table S6. Component distribution of the eICU-available reduced GIVP proxy**

**Supplementary Table S6A. Component-level distribution of the eICU-available reduced GIVP proxy**

| Component | Operational definition | Evaluable n | Positive n | Positive among evaluable, % | Not evaluable n | Positive among total cohort, % |
| --- | --- | --- | --- | --- | --- | --- |
| Absence of enteral nutrition initiation within 24 h | comp_en_not_started_24h: EN not initiated within 24 h of ICU admission | 319,503 | 309,950 | 97.0 | 0 | 97.0 |
| Delayed enteral nutrition initiation beyond 24 h | comp_en_delay_gt24h: EN was eventually initiated, but initiation occurred >24 h after ICU admission; evaluable only among patients with any recorded EN initiation. No positive events were observed in eICU-CRD. | 9,553 | 0 | 0.0 | 309,950 | 0.0 |
| Mechanical ventilation within 24 h | comp_mv_24h: Any mechanical ventilation within 24 h of ICU admission | 319,503 | 16,083 | 5.0 | 0 | 5.0 |
| Sedation within 24 h | comp_sed_24h: Any sedative agent administered within 24 h of ICU admission | 319,503 | 164,036 | 51.3 | 0 | 51.3 |

**Supplementary Table S6B. Reduced score distribution in eICU-CRD**

| Reduced score | n | Percentage of total cohort, % |
| --- | --- | --- |
| 0 | 3,126 | 0.98 |
| 1 | 152,541 | 47.74 |
| 2 | 153,980 | 48.19 |
| 3 | 9,856 | 3.08 |

**Supplementary Table S6C. Reduced GIVP high-risk distribution in eICU-CRD**

| Reduced GIVP high-risk status | n | Percentage of total cohort, % |
| --- | --- | --- |
| Reduced GIVP low-risk (reduced score = 0 or 1) | 155,667 | 48.72 |
| Reduced GIVP high-risk (reduced score ≥2) | 163,836 | 51.28 |

**Supplementary Table S6D. Hospital mortality according to reduced GIVP high-risk status in eICU-CRD**

| Reduced GIVP high-risk status | n | Hospital deaths, n | Hospital mortality, % |
| --- | --- | --- | --- |
| Reduced GIVP low-risk (reduced score = 0 or 1) | 155,667 | 11,996 | 7.71 |
| Reduced GIVP high-risk (reduced score ≥2) | 163,836 | 17,802 | 10.87 |

*Note: The eICU-available reduced GIVP proxy was constructed from four mappable early clinical indicators: absence of enteral nutrition initiation within 24 h, delayed initiation of enteral nutrition beyond 24 h, mechanical ventilation within 24 h, and sedation within 24 h. These indicators were coded as binary variables (comp_en_not_started_24h, comp_en_delay_gt24h, comp_mv_24h, comp_sed_24h) and summed to generate the reduced score. The delayed EN component (comp_en_delay_gt24h) was evaluable only in patients who received EN at any point (n = 9,553 of 319,503); 0 patients met the delay criterion, and this component contributed 0 to all scores in eICU-CRD. The maximum observed reduced score was therefore 3. Reduced GIVP high-risk was defined as a component sum ≥2. This table describes the component-level and score-level distribution of the reduced proxy in eICU-CRD and was used to improve transparency of the reduced external validation analysis. It should not be interpreted as full external reconstruction of the three-domain MIMIC-IV GIVP phenotype. Abbreviations: eICU-CRD, eICU Collaborative Research Database; EN, enteral nutrition; GIVP, gastrointestinal vulnerability phenotype; MIMIC-IV, Medical Information Mart for Intensive Care*
